# Disease Progression in *Plasmodium knowlesi* Malaria Is Linked to Variation in Invasion Gene Family Members

**DOI:** 10.1371/journal.pntd.0003086

**Published:** 2014-08-14

**Authors:** Atique M. Ahmed, Miguel M. Pinheiro, Paul C. Divis, Angela Siner, Ramlah Zainudin, Ing Tien Wong, Chan Woon Lu, Sarina K. Singh-Khaira, Scott B. Millar, Sean Lynch, Matthias Willmann, Balbir Singh, Sanjeev Krishna, Janet Cox-Singh

**Affiliations:** 1 Malaria Research Centre, University Malaysia Sarawak, Kuching, Sarawak, Malaysia; 2 School of Medicine, University of St Andrews, St Andrews, United Kingdom; 3 Faculty of Resource Science and Technology, University Malaysia Sarawak, Kuching, Sarawak, Malaysia; 4 Sibu Hospital, Sibu, Sarawak, Malaysia; 5 Sarikei Hospital, Sarikei, Sarawak, Malaysia; 6 Division of Clinical Sciences, St. George's, University of London, London, United Kingdom; 7 Clinical Blood Sciences, St. George's, University of London, London, United Kingdom; 8 Institute of Medical Microbiology and Hygiene, University of Tübingen, Tübingen, Germany; Institute of Tropical Medicine (NEKKEN), Japan

## Abstract

Emerging pathogens undermine initiatives to control the global health impact of infectious diseases. Zoonotic malaria is no exception. *Plasmodium knowlesi*, a malaria parasite of Southeast Asian macaques, has entered the human population. *P. knowlesi*, like *Plasmodium falciparum*, can reach high parasitaemia in human infections, and the World Health Organization guidelines for severe malaria list hyperparasitaemia among the measures of severe malaria in both infections. Not all patients with *P. knowlesi* infections develop hyperparasitaemia, and it is important to determine why. Between isolate variability in erythrocyte invasion, efficiency seems key. Here we investigate the idea that particular alleles of two *P. knowlesi* erythrocyte invasion genes, *P. knowlesi* normocyte binding protein *Pknbpxa* and *Pknbpxb*, influence parasitaemia and human disease progression. *Pknbpxa* and *Pknbpxb* reference DNA sequences were generated from five geographically and temporally distinct *P. knowlesi* patient isolates. Polymorphic regions of each gene (approximately 800 bp) were identified by haplotyping 147 patient isolates at each locus. Parasitaemia in the study cohort was associated with markers of disease severity including liver and renal dysfunction, haemoglobin, platelets and lactate, (r = ≥0.34, *p* = <0.0001 for all). Seventy-five and 51 *Pknbpxa* and *Pknbpxb* haplotypes were resolved in 138 (94%) and 134 (92%) patient isolates respectively. The haplotypes formed twelve *Pknbpxa* and two *Pknbpxb* allelic groups. Patients infected with parasites with particular *Pknbpxa* and *Pknbpxb* alleles within the groups had significantly higher parasitaemia and other markers of disease severity. Our study strongly suggests that *P. knowlesi* invasion gene variants contribute to parasite virulence. We focused on two invasion genes, and we anticipate that additional virulent loci will be identified in pathogen genome-wide studies. The multiple sustained entries of this diverse pathogen into the human population must give cause for concern to malaria elimination strategists in the Southeast Asian region.

## Author Summary

*Plasmodium knowlesi*, a parasite of Southeast Asian macaques, has entered the human population. Approximately 10% of *P. knowlesi* infections are severe, 1–2% are fatal, in Sarawak, Malaysian Borneo. Increase in parasitaemia is associated with disease severity, but little is known about parasite virulence in this newly described human pathogen. Here we present the results of a study on *P. knowlesi* parasites collected from 147 patients. We use the isolates to produce DNA sequences from a polymorphic (genetically variable) region of two *P. knowlesi* genes, *Pknbpxa* and *Pknbpxb*, that are involved in parasite entry into host red blood cells. We addressed the question that some parasite genotypes may have an invasion advantage leading to severe disease in human infections. We analysed the DNA sequences with matched clinical and laboratory data from the patient cohort (n = 147). We found that specific DNA sequences (*Pknbpxa* and *Pknbpxb* alleles) clustered with high parasitaemia and markers of disease severity. Here, for the first time, we provide evidence that variant alleles of the Plasmodium Reticulocyte Binding-Like Protein invasion gene family can influence disease progression in patients with malaria. The biological characteristics of the variants will be studied to aid our understanding of malaria pathophysiology and to inform intervention strategies.

## Introduction

*Plasmodium knowlesi* malaria is widespread in Southeast Asia (SEA). Descriptions of the aetiology of knowlesi malaria support a zoonotic origin of infection [1] and highlight variability in disease severity between those at risk across the region [2]. For example, very young children living in a forested area of Southern Vietnam have asymptomatic mixed *Plasmodium* species infections that include *P. knowlesi* [3]. Adults and children in Malaysian Borneo experience symptomatic single species *P. knowlesi* infections that are severe in >10% of patients and can be fatal [4], [5].

*P. knowlesi* transmission is restricted to the Leucosphyrus group of mosquito vectors found in forested areas of Southeast Asia [6], [7]. The vector group is diverse and capable of simultaneous transmission of human and non-human primate adapted *Plasmodium* species [8]. The majority of reported cases of *P. knowlesi* malaria are associated with time spent in the jungle or jungle fringe areas where the ranges of the natural vertebrate hosts, the long and pig tailed macaques (*Macaca fascicularis* and *Macaca nemestrina*) and leucosphyrus vectors overlap [9], [10]. However, a change in pattern has recently emerged in Malaysian Borneo, where children living in a deforested area are infected [11]. This new pattern may signal a change in vector or vector habitat preference and a move towards human-to-human transmission.

Restricted spread of *P. knowlesi* within human populations is attributed to non-urban vector habitat. Also human-host adapted Plasmodium species, where prevalent, may present a biological barrier to the entry of *P. knowlesi* into human populations concurrently at risk from human adapted and zoonotic species infections. On a backdrop of vector, human and parasite diversity, it would be folly for malaria elimination strategists to underestimate the importance of the multiple geographically dispersed entries of *P. knowlesi* into the human population [10]. The scene is set for a host switch of *P. knowlesi* from macaques to humans if pressed and as predicted by Garnham in 1966 [12].

Parasitaemia in malaria is a measure of the number of parasitized erythrocytes in the infected host at the time of sampling. The asexual replication cycle of *P. knowlesi* is 24 hours and therefore parasitaemia can increase daily in uncontrolled infections. Rising parasitaemia in *P. knowlesi* infections is associated with disease severity frequently involving renal failure, liver dysfunction and respiratory distress but not coma or severe anaemia [5], [13]. However, not all patients develop high parasitaemia, even following several days of untreated infection [5]. Parasite and/or host factors contributing to the rapid development of hyperparasitaemia in some patients infected with *P. knowlesi* have not been investigated.

Successful erythrocyte invasion by the infective merozoite stage of *Plasmodium* species is the result of a complex recognition, reorientation and entry process orchestrated by merozoite protein families conserved within the genus [14], [15], [16]. Of these, the reticulocyte binding-like protein (RBP) family is present in all Plasmodium species studied and members are involved in erythrocyte selection and invasion (Table S1). *P. falciparum* has five functional paralogous RBP members, PfRh1, PfRh2a, PfRh2b, PfRh4 and PfRh5 [17]. This family of proteins was first discovered in *P. vivax* [18], [19]. *P. vivax* has two well described functional members and more putative members have been identified recently [20]. The *P. falciparum* Rh proteins are thought to provide multiple invasion pathways allowing for invasion of a wide range of erythrocyte phenotypes, lessening restriction and explaining hyperparasitaemia [16], [21], [22]. There is also evidence for differential expression of the *PfRh* genes in human infections but with no clear association with parasitaemia and invasion efficiency [22], [23], [24]. *P. knowlesi* has two members of the RBP gene family, named *P. knowlesi* normocyte binding proteins *(Pknbp)xa* and *Pknbpxb* [25]. *Pknbpxa* is located on chromosome 14 and *Pknbpxb* on chromosome 7[26]. A recent study on an experimental line of *P. knowlesi* demonstrated binding of Pknbpxa but not Pknbpxb protein products to human erythrocytes implicating Pknbpxa in human erythrocyte invasion [27].

Here we report on a prospective study of patients with *P. knowlesi* malaria. We confirm and then exploit the association between *P. knowlesi* parasitaemia with clinical and laboratory measures of disease progression. We then address the question that parasitaemia in naturally acquired human infections is associated with particular alleles of the *P. knowlesi* merozoite invasion genes *Pknbpxa* and *Pknbpxb*.

## Materials and Methods

### Ethics statement

This non-interventional study was approved by Medical Research and Ethics Committee, Ministry of Health Malaysia and the Ethics Committee Faculty of Medicine and Health Sciences, University Malaysia Sarawak. The study was approved to recruit patients 15 years and above with informed signed consent. Children (<15 years) were not recruited into the study.

### Study design and patient recruitment

Two recruitment sites were selected for the study. The first, Hospital Sarikei, serves four districts in the Sarikei Health Division, population size 133,572 (2012) and Hospital Sibu, a referral hospital serving the Rejang basin. Patients with microscopy positive all-cause malaria were recruited with consent by the attending healthcare professionals. Each patient was given a unique study identifier code. Retrospective exclusion was based on PCR results. (For a detailed description of patient recruitment see Text S1)

### Blood sample processing

Serum, plasma and whole blood samples were stored frozen on site and transported at sub-zero temperatures to the Malaria Research Centre, University Malaysia Sarawak, (UNIMAS) at regular intervals during the study. DNA amplification, cloning and sequencing were conducted in UNIMAS and serum and plasma samples were shipped on dry ice to St George's University of London for glucose, lactate and IL-10 assays (See Text S1).

### PCR confirmation of *Plasmodium* species

DNA was extracted from dried bloodspot samples using the InstaGene method [28] to confirm the infecting parasite species. Nested PCR of the small subunit rRNA gene as described previously was used as follows: The first nest used primer pairs rPlu 5 and rPlu6 [29] and the second nests were specific for *P. falciparum*, *P vivax* [Paul C Divis, unpublished], *P. malariae* and *P. knowlesi* [1], [30].

### Data collection

A study dataset containing patient demographic information, history, clinical and laboratory information was prepared from the study history sheet and patient case notes using FileMaker Pro 10v.1 (FileMaker Inc.). Alleles occurring at the two genetic loci were added to the dataset during the course of the study. See below.

### De-selection of patients for the genotyping study

There were 232 patients with PCR confirmed single species *P. knowlesi* infections who fulfilled the study criteria (Figure S1a). Of these, 165 (71%) had mild disease with no abnormal clinical or laboratory indicators [5], [31]. In order to avoid generating redundant information and incur unnecessary costs these patients were sorted by parasitaemia and alternate patients were de-selected without biasing the range of parasitaemia in this group. Following de-selection, 147 patients were included in the genetic association study. This group included patients with mild malaria as above, all patients in the study with severe disease and all of those with some abnormal findings but with otherwise uncomplicated malaria using the WHO criteria for complicated malaria in the non-immune adult[31].

### Analyses for association between parasitaemia, clinical and laboratory markers of disease progression

Log transformed parasitaemia was normally distributed however some clinical and laboratory variables remained non-normally distributed. Spearman's (non-parametric) and Pearson's (parametric) correlation (*r*) were calculated Prism GraphPad v 4 and Stata 8 for Macintosh.

### *P. knowlesi* Pknbpxa and Pknbpxb reference DNA sequence

Pure DNA template was extracted from frozen EDTA whole blood samples using QIAamp DNA Mini kit (QIAGEN) following the manufacturer's instructions. *Pknbpxa* and *Pknbpxb* DNA sequence was generated from five reference *P. knowlesi* patient isolates using high stringency methodology as follows. Primer sequences were designed from published *Pknbpxa* and *Pknbpxb* sequences EU867791and EU867792 respectively [25]. In the first instance large segments of each gene (8501 bp *Pknbpxa* and 3506 bp *Pknbpxb*) were amplified and cloned as single fragments. The 8501 bp *Pknbpxa* fragment was amplified using the primer pair PknbpxaF5 5'AGGTGCAAGCTGGGAACAAG and PknbpxaR2 5'CTACACGACACACAATGCACC with LongRange PCR Enzyme Mix (QIAGEN). Each reaction was in a final volume 25 µL containing 2 µL DNA template, 0.4 µM each primer, 500 µM each dNTP and 1 U Long Range enzyme in 1x Long Range buffer (2.5 mM MgCl^2^). The reaction conditions were 93°C for 3 mins, 9 cycles at 93°C for 15 sec, 57°C for 30 sec, 68°C for 14 mins followed by 27 cycles at 93°C for 15 sec, 57°C for 30 sec and 68°C for 14 mins with addition of 20 sec/cycle. The *Pknbpxb* 3506 bp fragment was amplified using the primer pair Xb273F 5'GCATGGTCAAAAGAACCCC and Xb3430R 5'CTTCTATGGACGCTTCAGGT with Elongase Enzyme Mix (Invitrogen, Life Technologies). Each reaction was in a final volume of 20 µL containing 3 µL DNA template, 0.25 µM each primer, 500 µM each dNTP, 1 U Elongase in 1 x Elongase buffer (1.5 mM MgCl^2^) under the following conditions: 93°C 30 sec, 34 cycles at 93°C for 30 sec, 55°C for 30 sec, 68°C for 3 min followed by a final extension at 68°C for 10 mins. The fragments were cleaned, gel purified and cloned into the pCR TOPO XL vector, TOPO XL PCR Cloning Kit (Invitrogen, Life Technologies) following the manufacturer's instructions. Resulting plasmids were recovered using S.N.A.P. MiniPrep Kit (Invitrogen, Life Technologies). Each clone was sequenced in the forward and reverse directions beginning with M13F and M13R sequences flanking the insert followed by walk-in sequencing. Each sequencing reaction was performed in10 µL final volume with 2 µL BigDye Terminator v3.1 Cycle Sequencing (Applied Biosystems, Life Technologies), approximately 500 ng of plasmid with insert, 5 pmol primer with 35 cycles at 96°C for 20 sec, 50°C for 15 sec and 60°C for 4 minutes. Sequence reactions were ethanol/sodium acetate precipitated as per manufacturers instructions and outsourced to 1st BASE Pte Ltd (Malaysia) for sequencing. DNA sequences were aligned by CLUSTALW using MegAlign (Lasergene v7.0, DNASTAR). Two TOPO XL clones were generated from independent PCR reactions for each gene per reference isolate. Within clone DNA sequence conflicts were resolved before aligning the sequence of each clone. Between clone conflicts per isolate were resolved using a third clone.

### Haplotyping patient isolates *(Pknbpxa and Pknbpxb)*

The five reference sequences for *Pknbpxa* (8501 bp) and for *Pknbpxb* (3506 bp) were aligned using CLUSTALW, MegAlign Lasergene v 7.0 (DNASTAR) and exported to DnaSP v5.10 to calculate Nucleotide diversity (π) [32], [33]. Polymorphic fragments approximately 1000 bp suitable for direct PCR sequencing were identified in each gene. The primer pair *Pknbpxa*F5 5'-AGGTGCAAGCTGGGAACAAG-3' and 7428R1 5'- GCCAAGTCCAAACTTTTCCC-3' was designed to amplify a polymorphic *Pknbpxa* 1184 bp fragment under the following conditions: 3.0 µL DNA template, 0.4 U Phusion High-fidelity DNA polymerase (Thermo Scientific), 0.25 µM each primer, 500 uM each dNTP, 1 x Phusion buffer (1.5 mM MgCl^2^) in 20 µL final volume. The cycling conditions were 98°C for 30 sec and then 38 cycles at 98°C for 7 sec, 64.8°C for 20 sec and 72°C for 36 sec, followed by a final extension at 72°C for 10 minutes. Samples that failed to amplify were repeated with a new forward primer Ex1F 5'-GGTCCAAGAAATGTGCAAATG-3' designed from the chromosomal fragment Pk_strainH_chr14, correct at the time of the study (PlasmoDB [26]). The fragments were amplified using 3 µL DNA template, 1 U Elongase (Invitrogen life Technologies), 0.25 µM each primer, 500 µM each dNTP, 1 x Elongase buffer (1.5 mM MgCl^2^) in 20 µL final volume. Cycling conditions were: 93°C for 30 sec, 35 cycles at 93°C for 30 sec, 56°C for 30 sec and 68°C for 120 sec, followed by a final extension at 68°C for 10 minutes. The primer pair Xb272F and 3430R (as above) was used to amplify the *Pknbpxb* 3506 bp fragment in patient isolates as follows: 0.25 µM each primer; 4.0 µL DNA template; 500 µM each dNTP; 1 x Phusion buffer and 0.4 U Phusion DNA polymerase in 20 uL final volume. Cycling conditions were 98°C for 30 sec, then 38 cycles at 98°C 7 sec, 64.3°C 20 sec, 72°C 105 sec with a final step 72°C for 10 mins. Samples that were not amplified were repeated with the same primer pair and Elongase (Invitrogen Life Technologies) as per *Pknbpxb* amplification for cloning, table S2.

### Direct PCR sequencing

Fifteen µL of PCR products were cleaned with the PCR DNA fragments extraction kit, (Geneaid, Biotech Ltd.) as per manufacturer's instructions. Direct PCR sequencing was performed using 5 pmol primer (*Pknbpxa*F11 5'-TAAGCGAATCGAATAAGCAGCAG-3 for the *Pknbpxa* fragment and XB2318F 5'-GGTGTTCATGAAGATGTGCG-3' for the *Pknbpxb* fragment) with 2 µL BigDye Terminator v3.1 Cycle Sequencing (Applied Biosystems, Life Technologies). Between 34–36 ng of PCR template was included in 10 uL final volume reactions under the following conditions: 96°C 20 sec, 50°C 15 sec 60°C 4 min for 35 cycles. The reactions were Ethanol/Na acetate precipitated before sending to 1st BASE Pte Ltd (Malaysia) for sequencing as above. Only sequences with unambiguous base calls were included see table S2 which summarises PCR reactions and gives the number repeated, the number excluded and why. Sequences with two calls at particular sites, indicative of mixed genotype infections, were among those excluded.

### Nucleic acid sequence analyses

Nucleotide sequence diversity (π) was determined using DnaSP v5.10 software. A sliding window of 400 bases with 25 base steps was used when analysing the 8501 bp *Pknbpxa* fragment and 200 bases with step size 25 for the 3506 bp *Pknbpxb* fragment using DnaSP v5.10 software. Nucleotide diversity for the haplotyping fragments (approximately 800 bp in length) for both genes was determined on sliding window of 100 bases, with a step size of 10 bp. Parsimony informative sites, singleton sites, and the number of synonymous and non-synonymous substitutions were determined using DnaSP v5.10. The number of haplotypes (H) and haplotype diversity (Hd) for each locus was determined using DnaSP v5.10. A Minimum Spanning Haplotype Network was derived using Arlequin v3.5 [34]

### Genetic markers of disease progression

Continuous clinical and laboratory variables were tested for normality and those that failed the Kolmogorov-Smirnov test were log transformed. Outliers were identified using the interquartile rule. Values that were not within ±1.5 times the interquartile range were removed (Table S3). It should be noted that outliers were retained in the patient summaries (Table 1) and other analyses.

**Table 1:** Summary of demographic data, clinical characteristics and laboratory results for *P. knowlesi* patients [n = 232], *P. knowlesi* subset [n = 147], *P. vivax* and *P. falciparum* patients.

Variable [normal range]	*P. knowlesi*	*P. knowlesi* (study subset)	*P. vivax*	*P. falciparum*
*n* =	232	147	48	24
Age years - mean: std dev (range)	43.55∶14.61(16–84) [n = 231]	44.4∶14.6(16–84)	35.8∶10.87(15–62)*	34.8∶10.39 (18–52)*
Gender - % male	68	69	98*	88*
Axillary temperature - °C [normal range]	37.6(37.0–38.4)	37.6(37–38.5)	37.5(37–38)	38.4(37.3–39.00)*
History of fever - days	4(3–7) [n = 230]	4 (3–7)	5(3–7)	4(2.5–5.5)
Abdominal pain days mean (range)	1(0–14) [229]	1(0–7) [146]	0.5(0–7)	0.5(0–4)
Mean arterial BP-mmHg (mean/SD)[70 - 110]	87.5(12.31)[230]	86.86 (12.73)	87(14.18)	84.54(11.31)
Total parasitaemia/uL - geometric mean (IQR)	6993(1742–36734) [222]	10492(2090–51684) [143]*	2371(770–7296) [47] *	20,613(4030–76608)[23]*
Haemoglobin - g/dL [11.3–17.5]	13.6(12.1–14.7)[229]	13.4(12.3–14.7)	13.65(11.9–14.75)	13.5(12.2–14.5)
Platelets/uL [150,000–450,000]	58,500(37,000–89,000) [230]	55,000 (32,000–88,000)	85,000 (54,000–122,000)[47] *	70,500(42,000–116,500)
RBC ×10^6^/uL [4.2–6.1]	4.67(4.31–5.1)[182]	4.63(4.29–5.1) [109]	4.69(4.26–5.09)[37]	4.47(3.71–4.99)[14]
Packed Cell Volume (%) [36]–[52]	40(36–43)[221]	40(36–43) [139]	40.25(35.00–43.4)[46]	38(34.5–41.25)[21]
Leucocytes/uL [3.1–10.3]	6200(5000–7700)[229]	6350(5100–7900) [146]	5700(4550–6850)*	5100(4250–7300)
Neutrophils % Mean (std dev)	N/A	71.21 (8.086) [112]	N/A	N/A
Plasma Lactate - mmol/L [<2.0]	N/A	1.62(1.22–2.26) [111]	1.57(1.07–1.98)[31]*^§^	1.655(.91–2.055)[18]
Serum glucose - g/L [4.0–8.0]	N/A	6.35(5.57–7.23)	6.47–5.32–8.23[31]	5.92(4.9–6.73)[18]*^§^
Sodium mmol/L [136–152]	133-130-136)[225]	133(130–136) [143]	136(134–138)[47] *	132(129–136)[23]
Total Bilirubin - umol/L [<21.0]	21.2(14.1–35.4) [214]	23.1(15.75–47.65) [130]	17.3(11.5–26.9)[41] *	27.0(17.1–55.1)[23]
Conjugated bilirubin - umol/L [<1.7]	8.3(4.1–13.6)[203]	8.65(4,5–20.9) [130]	5.8(3.9–11.4)[39]	11.15(5.65–29.1)[22]*
Alanine aminotransferase U/L [<40]	40(25–64)[213]	39 (25–63) [135]	30(21–52.5)[45] *	38(20–54)[23]
Aspartate aminotransferase U/L [<37]	37(25–61)[213]	42(28–63) [135]	37(22–47.5)[45] *	32(25–73)[23]
Alkaline phosphatase [39–117]	109(77.5–165.5)[213]	111(82–170)[135]	86(65.5–123.5)[45] *	88(55–120)[23]*
Serum creatinine - umol/L [<133.0]	89(75–117) [199]	95(80–134) [131]	97(81–110)[43] *	99(87 –112)[23]
Blood Urea mmol/L [1.0–8.3]	5.7(4.3–8.8)[223]	6.3(4.5–11.74)[141]	4.7(3.9–6.6)*	5.0(4.0–8.1)[23]
Total Protein g/ [61–76]	69(64–74.5)[212]	69(64–74)[135]	70(64–74)[[45]	63.5(59–70.5)[22]*
Serum Albumin g/L [>36]	37(32–40)[212]	36(31–39)[135]	36(32–41)[45]	34(29.5–38)[22]
Serum globulin g/L [23]–[35]	32(29–36) [199]	32(29–36) [131]	31 (27.5–34.5) [45]	32(26–33.5) [21]

Pre-treatment values and measurements are summarised as median (inter quartile range) except where stated differently. Numbers in square brackets, []  = n when different from total number of patients in each group. The *P. knowlesi* [147], *P. falciparum* and *P. vivax* groups were tested for significant differences with the *P. knowlesi* [232] group using the Mann-Whitney U Test and the un-paired t test with Welch's correction, Prism 4 for Macintosh, GraphPad Software, Inc. * *p* = <0.05 compared with *P. knowlesi* [232] and **^§^**when compared with *P. knowlesi* [147].

To identify the haplotypes groups, in *Pknbpxa* and *Pknbpxb* genes, a systematic method was applied to count the number of variations in each position and aggregate those with same number of occurrences. Only positions with mean allelic frequency >12 % were considered. The assigned groups covered most of the possible allelic combinations (Table S4).

*Pknpbxa* and then *Pknbpxb* haplotyping sequences from each patient isolate were aligned and polymorphisms with minimal allelic frequency (MAF) >12% were added to the patient dataset. The method above was applied to identify haplotype combination groups each with 2-3 alleles for *Pknpbxa* and *Pknpbxb* (Table S4). All data, clinical, laboratory and allelic groups were combined and imported into Prism GraphPad v 4 for graphical representation and tests for significant differences. In order to validate our results thirteen computer-generated randomized datasets were created. For this the patient clinical and laboratory data (23 variables) were assigned the haplotype data in a random fashion and the randomization repeated 12 times. We then performed 't' tests on the 299 possible combinations (23×13) to detect statistically significant clustering within the random groups. By applying confidence limits of 95%, 14.95 combinations out of 299 would be expected to be significant by chance alone. We detected only 8 random events. Therefore it is highly unlikely that our findings could be the result of chance or statistical artifact.

All variables were tested independently even when likely to be on the same causal pathway. Based on the Minimum Spanning Haplotype Network method, Arlequin v3.5 [34], the haplotype groups were mapped onto the minimum spanning network by applying the analysis of molecular variance (AMOVA). The networks were resolved first with the Gephi v0.8.2 Beta [35] and manually edited to include missing mutations, Arlequin v3.5, to connect each haplotype identified in the study.

In addition association between parasitaemia and relevant variables were adjusted for potential confounders by multivariate linear regression using a stepwise backward elimination process using Stata version 12.0 (Stat Corp., College Station, TX, USA). Variables associated with parasitaemia in a univariate analysis (p<0.1) were included in the multivariate models. *p* = <0.05 was required to retain variables in the final model. Data were transformed when heteroskedasticity was detected (Cook and Weisberg's test).

### Linkage disequilibrium

Linkage disequilibrium (LD) was inferred with Haploview [36] and the data were transformed to be loaded with the X chromosome format. Both *Pknbpxa* and *Pknbpxb* haplotypes per patient isolate were analysed as a continuum to deduce LD within and between them.

## Results

### Patient cohort

Of 389 patients admitted into the study between January 2008 and February 2011, 304 had PCR-confirmed single species *Plasmodium* infections: 232 (76%) *P. knowlesi*, 24 (8%) *P. falciparum* and 48 (16%) *P. vivax* (Text S1 and Figures S1a and S1b). Demographic information with pre-treatment, clinical and routine laboratory results for all three species infections are summarised in table 1. *P. vivax* and *P. falciparum* data are included for comparison. Eighty-five *P. knowlesi* patients were deselected (see below and materials and methods section) creating a subset of 147 *P. knowlesi* patients for *Pknbpxa* and *Pknbpxb* genotyping.

Clinical, demographic and laboratory data of the *P. knowlesi* subset [n-147] were representative of the total number of knowlesi patients [n = 232] with the exception of parasitaemia (Table 1). The subset of patients [n = 147] had a significantly higher geometric mean parasitaemia 10492 (2090-51684) parasites/uL (IQR) compared with 6993 (1742-36734) in the larger group, *p* = 0.03 (Mann Whitney U test). Parasitaemia was not used as a criterion for de-selection but a shift in parasitaemia was expected when approximately 50% of the relatively large group of patients with mild malaria (no abnormal clinical or laboratory results) were randomly removed. Forty-six (46) women and 101 men remained in the subset (n = 147). Women had a higher pulse rate, lower PCV and haemoglobin than men but this is unlikely to be related to their infection (Figure S2). The following variables; conjugated bilirubin (*p* = 0.021, total protein (*p* = 0.001), serum albumin (*p* = 0.031) and serum globulin (*p* = 0.001) all associated with recruitment hospital site, probably due to different measuring systems, and were removed from further analyses.

### Association between parasitaemia, clinical and laboratory markers of disease progression

The clinical and laboratory variables (Table 1) were analysed individually for association with parasitaemia (Table 2 for the n = 147 subset and Figure S3 for the n = 232 complete *P. knowlesi* cohort). Parasitaemia in both groups was associated with plasma lactate, serum creatinine and blood urea (*r* = >0.50; *p* = <0.0001) and with haemoglobin, neutrophils, platelets, total bilirubin, AST and sodium levels (*r* = >0.33 and <0.50, *p* = <0.0001).

**Table 2:** Clinical and laboratory measures of disease progression that associate with *P. knowlesi* parasitaemia.

Variable - association with parasitaemia	n =	(r)	*p* value
Age in years	143	+0.274*	0.0009
Haemoglobin g/d:	142	−0.366	<0.0001
PCV (%)	135	−0.282	0.0009
Log_10_ WBC/uL	143	+0.444	<0.0001
% Neutrophils	108	+0.392	<0.0001
Log_10_ Platelets/uL	143	−0.327*	<0.0001
Log_10_ Lactate mmol/L	110	+0.501	<0.0001
Log_10_ Serum creatinine umol/L	129	+0.528	<0.0001
Log_10_ Blood urea mmol/L	137	+0.601	<0.0001
Log_10_ Total bilirubin umol/L	128	+0.403*	<0.0001
Log_10_ Conjugated bilirubin umol/L	128	+0.330	<0.0001
Log_10_ AST U/L	132	+0.355*	<0.0001
Log_10_ IL-10 pg/mL	142	+0.362	<0.0001
Sodium mmol/L	139	−0.328	<0.0001

The (r) statistic was calculated using Prism 4 for Macintosh, GraphPad Software, Inc. Pearson's correlation was used for parametric data and marked* otherwise for non-parametric data Spearman's correlation test was used.

### *P. knowlesi Pknbpxa* and *Pknbpxb* reference sequences

**Figure 1:**
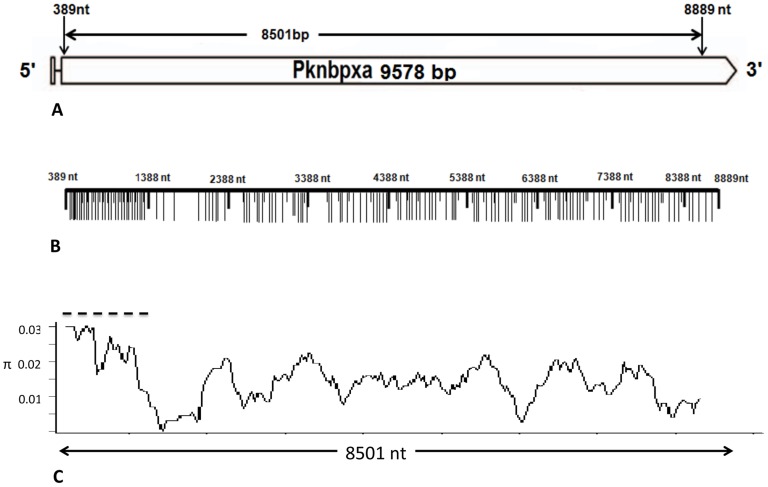
*P. knowlesi Pknbpxa* organisation and diversity. Schematic representation of *Pknbpxa* 9578 bp. (**A**) Exon 1 and the intron (solid line) are followed by exon II begining at nucleotide 389 (EU867791). Pknbpxa cysteine residues at codon positions 181,239,283,311 and 315 that are implicated in erythrocyte binding, Meyer, *et al.*, [23], were within the haplotyping fragment and conserved in all patient isolates. (**B**)A fragment from nucleotide 389–8889 (8501bp) was amplified and sequenced in five reference isoates. Synonymous (short vertical lines) and non-synonymous (long vertical lines)mutations are marked. (**C**) Graphical representation of a sliding window plot of nucleotide diversity per site. Diversity (**π**) was calculated using DnaSP v5.10 with window length 400 bp and step size 25 bp. Maximum diversity (**π** = 0.024) was observed between nucleotide positions 389 and 1388 (hatched line).

**Figure 2:**
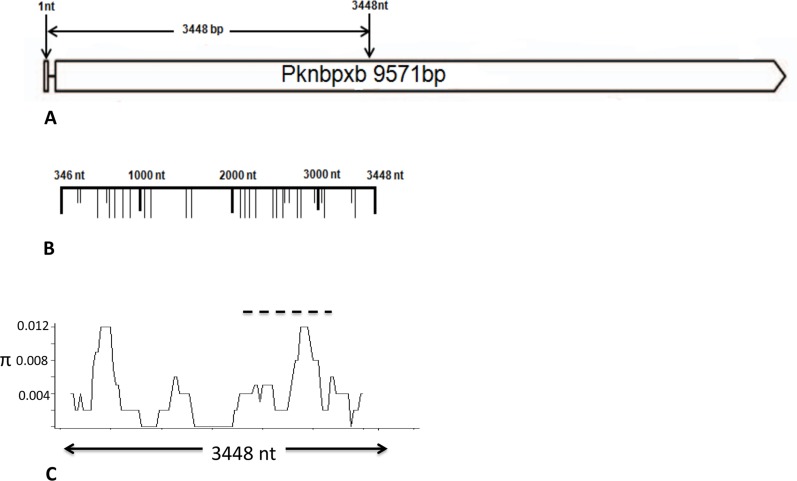
*P. knowlesi Pknbpxb* organisation and diversity. Schematic representation of *Pknbpxb* 9571 bp. (**A**) Exon 1 and the intron (solid line) are followed by exon II beginning at nucleotide 346 (EU867792). Pknbpxb cysteine residues at codon positions 193,254,298,326 and 332 that are implicated in erythrocyte binding, Meyer *et al*., [23], were not within the haplotyping fragment but were conserved in the five patient reference isolates. (**B**) A fragment from nucleotide1 to 3448 was amplified in five reference isolates. Synonymous (short vertical lines) and non-synonymous (long vertical lines)mutations are marked. (**C**) Graphical representation of a sliding window plot of nucleotide diversity per site. Diversity (**π**) was calculated using DnaSP v5.10 with window length 400 bp and step size 25 bp. Maximum diversity (**π** = 0.0056) was observed between nucleotide positions 2275 to 3156 (hatched line).

Local *Pknbpxa* and *Pknbpxb* diversity was determined by sequencing, to high stringency, large fragments of both genes from five *P. knowlesi* isolates collected in different locations and times (Table S5). *P. knowlesi Pknbpxa* (8501 bp) comprising all but the first 18 and last 689 bases of exon II was amplified, cloned and sequenced (Figure 1a) and *Pknbpxb* (3506 bp) comprising exon I, the intron and 3102 bp of exon II (Figure 2a).

*Pknbpxa* reference sequences were aligned with the published sequence from the *P. knowlesi* experimental H line EU867791. There were no gaps in the alignment and phylogenetic analysis indicated that the *Pknbpxa* gene was dimorphic (Figure S4). The 8501 bp *Pknbpxa* gene fragment was polymorphic (nucleotide diversity, π = 0.0142±0.0029, n = 5) and comprised 168 non-synonymous and 63 synonymous polymorphic sites (Figures 1a-c and Table 3). There was a cluster of non-synonymous SNP's from base 389 to 1388 (Figure 1c) and this region was selected for direct PCR sequencing and haplotyping 147 patient isolates at the *Pknbpxa* locus. The *Pknbpxa* reference sequences were submitted to GenBank, Accession Numbers KF186568-KF186572, (Table S5).

**Table 3:** *Pknbpxa* and *Pknbpxb* reference and haplotyping sequence diversity.

	n	BP	SNP	NS	S	π (SD)	d (SE)
*Pknbpxa* (Reference)	5	8501	231	168	63	0.0142 (0.00287)	0.0145 (0.0011)
*Pknbpxa* (Haplotyping)	138	885	83	57	26	0.0232 (0.00029)	0.0233 (0.0034)
*Pknbpxb* Reference	5	3100	28	20	8	0.00382 (0.00121)	0.0038 (0.0007)
*Pknbpxb* Haplotyping	134	879	46	27	19	0.00639 (0.00024)	0.0064 (0.0015)

n = number of sequences sampled; BP = number of sites analyzed (all within coding region and there were no gaps); SNP = number of polymorphic sites; NS = number of non-synonymous substitutions; S = number of synonymous substitutions; **π** = average pairwise nucleotide diversity calculated using Jukes-Cantor correction with standard deviation in parenthesis calculated using DnaSP v 5.10.01; d = nucleotide diversity calculated using Tamura's three-parameter model with standard error in parentheses calculated using MEGA v 5.05

*Pknbpxb* reference sequences, comprising exon I to midway through exon II, were generated (Figure 2a). Due to DNA polymerase slippage while sequencing through the "AA" homo polymeric region towards the 5' end of exon I and also due to "AT" tandem repeats within the 243 bp intron the sequences were trimmed to 3100 bp, removing exon I and the intron. Alignment of the five 3100 bp reference sequences with the published *Pknbpxb* sequence from *P. knowlesi* H (EU867792) showed that the reference sequences had three single nucleotide deletions at positions G2477, A2478 and C2488 relative to EU867792. The deletions did not introduce stop codons, however translation into amino acid sequence identified an additional amino acid position in the published Pknbpxb protein sequence (ACJ54536). This resulted in a four amino acid motif (GKIY) unique to the published sequence that differed from a conserved three amino acid motif (ENL) in patient isolates (Figure S5). The *Pknbpxb* reference sequences were submitted to GenBank,

Accession Numbers KF186573-KF186577, (Table S5).

There were 20 non-synonymous and 8 synonymous sites between the five *Pknbpxb* reference sequences with nucleotide diversity **π** = 0.00381±0.0007 (Figure 2 a - c and Table 3). The highest concentration of non-synonymous SNPs was between bases 2275 and 3156 (Figure 2c) and this region was chosen for haplotyping patient isolates.

### *Pknbpxa* and *Pknbpxb* direct PCR sequencing patient isolates

Of 147 patient isolates 138 *Pknbpxa* (885 bp) and 134 *Pknbpxb* (879 bp) sequences were generated by direct PCR sequencing. *Pknbpxa* sequencing results were not obtained for seven patient isolates and two isolates had unacceptable sequencing readouts (Table S2). Three patients appeared to have mixed *Pknbpxb* genotype infections, seven isolates failed to amplify in each of *Pknbpxb* PCR reactions and three isolates had poor sequence outputs (Table S2).

### *Pknbpxa*

**Figure 3:**
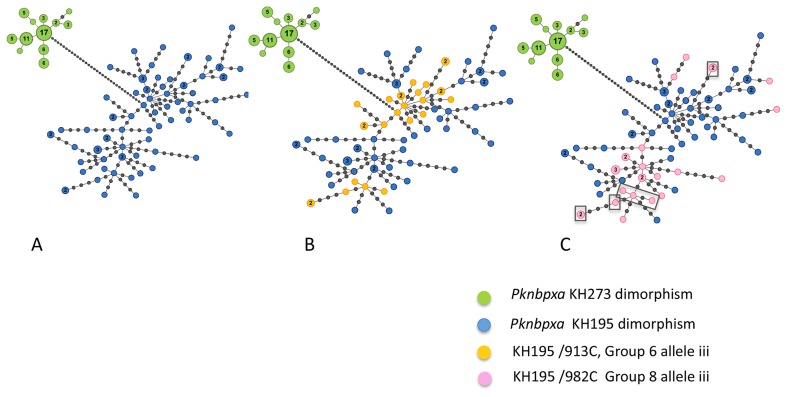
*Pknbpxa* minimum spanning haplotype network. (**A**) 75 haplotypes were resolved in 138 patient isolates coloured nodes. Isolates in the KH273 dimorphic group are in green and those in the KH195 dimorphism in blue. Each node represents one haplotype and the size of the coloured nodes is relative to the frequency. The frequency number is given for all nodes with a frequency >1. Intermediary gray nodes represent missing haplotypes required to connect two different haplotypes. (**B**) Haplotypes with *Pkbnpxa* group 6 allele iii (913C) that had increased markers of disease severity are shown in yellow. *P. knowlesi* isolates with this mutation appear in 2 clusters within the KH195 dimorphism. (**C**) Haplotypes with *Pknbpxa* group 8 982 alleles are shown: 982T allele i (KH273 green); 982G allele ii (KH195 blue); 982C allele iii (KH195 pink). Group 8 alleles ii and iii had increased markers of disease severity when compared with allele i. There is one main cluster of 982C (pink) haplotypes with 5 additional and apparently un-connected to the main cluster that appear on the edges of the network. 982C (pink) haplotypes all occur in the KH195 dimorphic group (4a blue). Note that the boxed nodes also contain *Pknbpxa* 913C (4b). Haplotypes were generated using Arlequin v3.5.1.2 and the network drawn with Gephi v0.8.2 with manual editing to add the missing haplotypes. Haplotype groups were mapped onto the minimum spanning network by applying the analysis of molecular variance (AMOVA).

Gaps or deletions were not detected when the 138 *Pknbpxa* 885 bp sequences were aligned. There were 83 (9.4%) polymorphic sites and 802 (90.6%) invariant sites (Table 3). Sixty-two (74.6%) of the polymorphic sites were parsimony informative. Phylogenetic and network analyses showed a clear dimorphism involving a 29 base haplotype with 25 non-synonymous and 4 synonymous changes (Table S6). Seventy-seven (56%) isolates clustered in the KH195 dimorphic group and 61 (44%) formed the KH273 dimorphic cluster (Figure 3A). The Pknbpxa amino acid changes relative to the published reference sequence ACJ54535 are given in table S7a.

Seventy-five (75) *Pknbpxa* DNA haplotypes were resolved in the 885 bp fragments from 138 patient isolates with haplotype diversity 0.9729 (SD±0.007). There were 12 (16%) haplotypes in the KH273 dimorphic cluster all but four with a frequency >2 and 63 (84%) haplotypes in the KH195 cluster. All but 11 of the Pknbpxa KH195 haplotypes had a frequency <1 (Figure 3A).

### *Pknbpxb*

**Figure 4:**
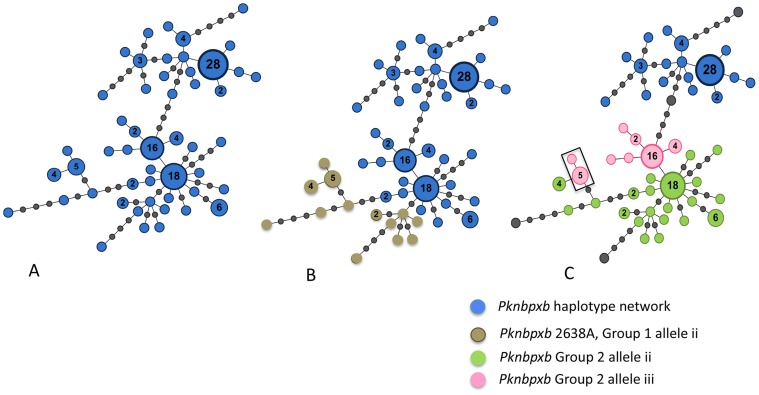
*Pknbpxb *minimum spanning haplotype network. (**A**) 51 haplotypes were resolved in 134 patient isolates (Blue). Each node represents one haplotype and the size of the coloured nodes is relative to the frequency. The frequency number is given for all nodes with a frequency >1. Intermediary gray nodes represent missing haplotypes required to connect two different haplotypes. (**B**) *Pknbpxb* haplotype group 1. Haplotypes with allele ii *Pkbnpxb*2638A, lower haemoglobin and higher axillary temperature, are shown in brown radiating from a high frequency haplotype (f = 18). (**C**) *Pknbpxb* group 2 haplotypes, alleles i (blue), ii (green) appeared as discrete clusters Allele iii (pink) had increased markers of disease severity and formed 2 clusters. Four isolates were excluded and appear as larger grey nodes. Haplotypes with alleles ii and iii cluster together. Alleles shared with *Pknbpxb* group 1 are boxed. Haplotypes were generated using Arlequin v3.5.1.2 and the network drawn with Gephi v0.8.2 and manual edited to add the missing haplotypes markers. Haplotype groups were mapped onto the minimum spanning network by applying the analysis of molecular variance (AMOVA).

The three gaps observed in five *Pknbpxb* reference sequences were conserved in all patient isolates (n = 134) in the study relative to the published sequence *Pknbpxb* EU86772. Alignment of the 134 *Pknbpxb* 879 bp sequences identified 46 polymorphic sites (Table 3) and 25 of the sites were parsimony informative. Fifty-one (51) *Pknbpxb* haplotypes were resolved with haplotype diversity  = 0.922 (SD±0.014). A minimum spanning haplotype network shows some dimorphic characteristics at the *Pknbpxb* locus, Figure 4A. Pknbpxb amino acid changes relative to the published reference sequence ACJ54536 are given in table S7b.

### Genetic markers of disease progression

Twelve *Pknbpxa* and two *Pknbpxb* haplotype groups were resolved, each with two or three allelic forms. The alleles were added to the patient dataset and analysed for significant differences in clinical and laboratory markers of disease severity (Table S4). *Pknbpxa* alleles that segregated with markers of disease severity were within the KH195 dimorphism. For example patients infected with parasites with *Pknbpxa* group 6, 913C allele iii were restricted to the KH195 dimorphism and had; higher parasitaemia (*p* = 0.02), WBC's (*p* = 0.02), blood urea (*p* = 0.003), serum creatinine (*p* = 0.0057), plasma lactate (*p* = 0.003), IL-10 (*p* = 0.003), lower platelets (*p* = 0.001), systolic blood pressure (*p* = 0.005) and prolonged duration of fever (*p* = 0.036) (Figure 3B and Figure S6 a-j). When isolates with the 913C polymorphism were overlaid on the haplotype network a degree of haplotype clustering was observed although the allele appeared in two distinct nodes (Figure 3B). *Pknbpxa* haplotype group 8 describes a complex polymorphic site (982T/G/C). Parasites in the KH273 dimorphism all had 982T allele i (n = 61). The KH195 dimorphism had two alleles, allele ii, 982G (n = 51) and allele iii, 982C (n = 26). Alleles ii and iii had some association with markers of disease progression. Patients infected with parasites with 982G allele ii had higher plasma lactate (*p* = 0.006), higher IL-10 (p = 0.03) and lower haemoglobin (*p* = 0.034) and those with 982C allele iii had higher AST (*p* = 0.033), (Figure 3C and Figure S7 a-f). When the 982C and 982G, group 8 alleles ii and iii were mapped onto the *Pknbpxa* haplotype network they formed clusters. However five haplotypes with group 8 allele iii appeared on the edges of the group 8 allele ii cluster (Figure 3C). There was some overlap between *Pknbpxa* groups 6 and 8 (Figures 3C boxed area).

Two *Pknbpxb* allelic groups were resolved. *Pknbpxb* group 1 had two alleles, 2637A:2638C and 2637C:2638A. Patients infected with parasites with the 2637C:2638A allele had lower haemoglobin (*p* = 0.004) and higher axillary temperature (*p* = 0.001) (Figure 4B and Figure S8a and S8b). Isolates with 2637C:2638A appeared as two clusters at the edges of the *Pknbpxb* haplotype network (Figure 4B). *Pknbpxb* group 2 was more complex with three major alleles (i, ii, iii) involving 6 polymorphic sites (Table S4). Four patient isolates were not easily grouped and excluded from the *Pknbpxb* group 2 analysis. Patients infected with parasites with *Pknbpxb* group 2 allele iii had increased parasitaemia (*p* = 0.016), plasma lactate (*p* = 0.004), total bilirubin (*p* = 0.047) and AST (*p* = 0.002), Figure 4B and Figure S9a-f. Haplotype clustering was observed when *Pknbpxb* group 2 alleles i, ii and iii were mapped onto the *Pknbpxb* haplotype network (Figure 4C). *Pknbpxb* group 2 allelic cluster i was not associated with markers of disease progression and formed a discrete cluster in the *Pknbpxb* network. Group 2 allele ii also formed a discrete cluster and allele iii appeared as 2 clusters. In addition there was allelic sharing between *Pknbpxb* haplotype group 1 allele ii and Group 2 allele iii (Figure 4C boxed).

### Linkage disequilibrium within and between *Pknbpxa* and *Pknbpxb* sites

**Figure 5:**
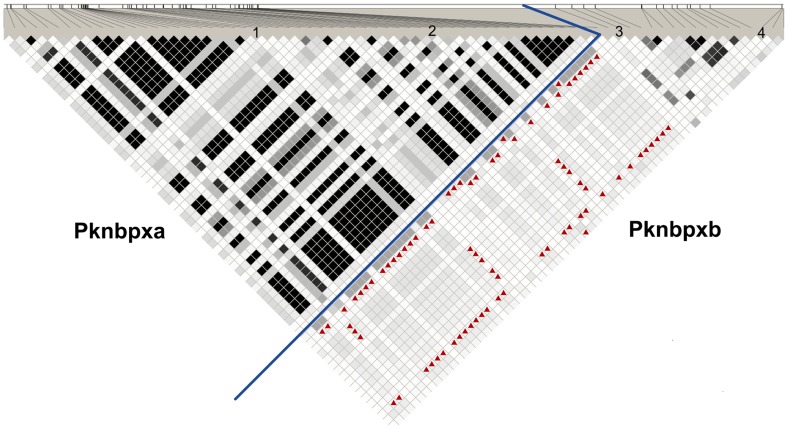
Linkage disequilibrium (LD), *Pknbpxa* and *Pknbpxb*. The LD matrix was inferred with Haploview (Barrett et al, 2005) and an in-house script for data input in the X chromosome format suitable for haploid data. *Pknbpxa* alleles are to the left of the blue line and *Pknbpxb* to the right. The intensity of shading reflects the strength of linkage in the correlation between pairs of loci (r^2^), black being strong r^2^ >0.8. Linkage between the two genes was detected between *Pknbpxa* positions 810 and 1105 marked1 and 2 respectively and *Pknbpxb* positions 2403 and 3110 marked 3 and 4 respectively. Linkage between these sites is shown as red triangles where D'>0.99 and LOD>2 but with otherwise low r^2^ values.

A strict 29 bp haplotype defines the *Pknbpxa* KH195 - KH273 dimorphism in the *Pknbpxa* haplotyping sequence (885 bp). The intensity of r^2^ indicates that several *Pknbpxa* alleles, including those defining the dimorphism are in strong LD r^2^>0.8 forming linked blocks in the matrix (Figure 5). The r^2^ values for linked sites between the two genes were weak. However, there was some between gene linkage with high D' values (>0.99) and LOD>2 (Figure 5). For example *Pknbpxa* position 810 and *Pknbpxa* position 1105 (Figure 5, positions marked 1 and 2. Also *Pknbpxb* positions 2403 and 3110 with the main blocks defining the *Pknbpxa* dimorphism (Figure 5 positions marked 3 and 4).

## Discussion

Patients with *P. knowlesi* malaria in Sarawak are infected with diverse parasites at the *Pknbpxa* and *Pknbpxb* loci. Some of the diversity clusters with increased parasitaemia and measures of disease severity. To our knowledge this is the first time that particular alleles of members of the *Plasmodium* RBP gene family have been linked to increased parasitaemia and disease in patients with malaria.

Members of the RBP's are represented in all Plasmodium species studied so far [17]. They are diverse in nature and thought to be responsible for erythrocyte selection during merozoite invasion of host erythrocytes. Even though humans are not the natural hosts of *P. knowlesi* we did not find evidence of human host selection, represented by clonality at these important loci. In agreement with *P. falciparum* (*Pf*) *Rh1, PfRh2a, PfRh2b* and *P. vivax RBP-2* non-synonymous substitutions were predominant at each *P. knowlesi* RBP locus [37]. The 5' end of *Pknbpxa*, corresponding to the putative erythrocyte-binding site, was particularly polymorphic although polymorphic sites occurred along the entire 8105 bp fragment, similar to *P. vivax RBP-2* [37].

Diversity involving non-synonymous substitutions near functional sites on *Plasmodium* merozoite surface proteins is not unusual and a particular problem in vaccine design [38], [39]. Non-synonymous diversity is most often attributed to immune evasion rather than altered function. Although a single amino acid change in experimental lines of *P. falciparum* reticulocyte binding-like orthologue *PfRh5* changed function and conferred infectivity to Aotus monkey erythrocytes [40]. Therefore, assigning loss of host erythrocyte restriction to a single amino acid suggests that non-synonymous substitutions on other members of this invasion gene family may alter function as well as antigenicity. Patients in our study were infected with *P. knowlesi* parasites with predominantly non-synonymous substitutions at the RBP invasion gene loci. This observation will be taken forward to examine altered protein function between Pknbpxa and Pknbpxb protein variants and the impact of variability on parasite virulence and host invasion restriction.

We did not observe parasite clonality in *P. knowlesi* infections that would suggest restricted entry of *P. knowlesi* genotypes into the human host at this or other loci [1]. However, approximately half (44%) of the patients in our study were infected with *Pknbpxa* KH273 parasites, comprising only 12 haplotypes, and we cannot rule out some selection in the absence of *Pknbpxa* and *Pknbpxb* sequence data from the natural macaque hosts. Highly relevant to our study, children in Ghana infected with *P. falciparum* isolates with the CAMP dimorphism of EBA-175, a member of another important Plasmodium invasion gene family, were at higher risk of a fatal outcome [41]. In our study *Pknbpxa* alleles that clustered with increased markers of disease progression were within the *Pknbpxa* KH195 dimorphism. Polymorphisms within the CAMP dimorphism were not interrogated in the Ghanaian study and this approach may have identified putative virulent haplotypes. However, taken together with our results, characterising within species differences in parasite virulence may provide insight into the potential health impact of malaria, especially during outbreaks.

The relationship between *P. knowlesi* parasitaemia and markers of disease severity is consistent with other published studies [4], [5], [42], [43]. High parasitaemia was not associated with prolonged duration of symptoms in this or other studies suggesting parasite or host specific reasons for the apparent increase in rate of parasitaemia in some patients [5], [42], [44]. Here we show that parasitaemia was significantly higher in patients infected with parasites with *Pknbpxa* group 6, 913C allele iii or *Pknbpxb* group 2 allele iii. Patients infected with the *Pknbpxa* allele had some but not all of the markers associated with parasitaemia in this study. For example, they had significantly higher markers of renal dysfunction but not liver dysfunction or significant changes in haemoglobin or PCV. What at first consideration may have suggested a phenotypic effect of increased invasion efficiency and rapid progression to high parasitaemia may well be the opposite. Patients infected with this variant had higher parasitaemia and longer duration of symptoms. It is possible that a prolonged progression to high parasitaemia may impact pathology. It is worth noting that these patients also had increased lactate and lower systolic blood pressure. In addition to higher parasitaemia, patients infected with *Pknbpxb* group 2 allele iii had increased total Bilirubin, AST and plasma lactate. Taken within the important caveat that parasitaemia is central to our study and in multivariate linear regression models parasitaemia is independently associated with PCV, AST (transformed data), urea and plasma lactate (transformed data), there was some stratification between the disease markers that clustered with the particular *Pknbpxa* and *Pknbpxb* alleles. Patients infected with parasites with *Pknbpxb* group 1 allele ii, 2637C:2638A had lower haemoglobin and higher axilliary temperature but not higher parasitaemia. The *Pknbpxa* alleles but not *Pknbpxb* were associated with markers of renal dysfunction. To our knowledge this is the first report of an association between specific invasion gene alleles and markers of disease progression, including parasitaemia, in malaria even though hyperparasitaemia is one of the WHO criteria for severe malaria in *P. falciparum* and *P. knowlesi* infections.

Purified *P. knowlesi* H Pknbpxb protein did not bind to human erythrocytes under experimental conditions calling into question the role of this protein in human infections [27]. However, here, patients infected with particular *P. knowlesi Pknbpxb* alleles had significantly different haemoglobin levels, axillary temperature, parasitaemia, PCV, lactate, bilirubin and AST. These altered disease phenotypes suggest that at least some *Pknbpxb* variants in nature are important in human infections. *Pknbpxb* was less diverse than *Pknbpxa* and there was some evidence of linkage between the two genes even though they appear on different chromosomes suggesting a degree of co-operation [26]. Co-operation between members of invasion gene families was reported in *P. falciparum* infections [45].

Patients infected with different allelic forms of *P. knowlesi Pknbpxa* and *Pknbpxb* had differences in markers of disease progression. Parasites with putatively virulent alleles formed two clusters on the *Pknbpxa* minimum spanning tree network with some overlap and were confined to the KH195 dimorphism. Putatively virulent *Pknbpxb* alleles were confined to two closely related clusters also with some overlap, a third *Pknbpxb* cluster was not associated with markers of disease progression. Genetic diversity, haplotype clustering and associated differences in markers of disease severity may explain the wide range of *P. knowlesi* disease phenotypes observed in communities across South East Asia.

As expected polymorphisms on small fragments representing <10% of two *P. knowlesi* genetic loci did not capture all patients with complications in our study. Therefore further work on full-length *Pknbpxa* and *Pknbpxb* gene sequences may improve the resolution of our study. Also it is expected that virulence would not be restricted to two genetic loci.

Historically, experimental lines of *P. knowlesi* have informed Plasmodium biology, including erythrocyte invasion [46]. More recently clinical studies on *P. knowlesi* malaria have added context to the study of severe malaria. Current advances in pathogen genome sequencing and application, to even small quantities of parasite DNA such as that available from our patient isolates, will allow us to extend our work on parasite virulence to multiple candidate loci genome wide. This, taken together with the recent publication of *P. knowlesi* culture adaptation to human erythrocytes and an efficient transfection system will provide the means to assign disease phenotype to genotype using rational and systematic experimental design not only *in vitro* but *in vivo* [47].

Information emerging from clinical and laboratory studies on *P. knowlesi* is new and pertinent to our understanding of malaria pathophysiology, including malaria caused by other Plasmodium species infections (Table 1) [5]. *P. knowlesi* is a zoonotic pathogen that is permissive in a variety of differentially susceptible non-human primates [48], [49], [50], [51], [52]. Surely there is a compelling argument to use *P. knowlesi* as a robust representative animal model for malaria pathophysiology. The lack of such a model so far has impeded proof of principle testing towards the rational development of new tools to treat and manage severely ill patients with malaria.

## Supporting Information

Figure S1 **Patients with malaria recruited into the study.** (**A**) Of 261 patients recruited with PCR-confirmed single species *P. knowlesi* (Pk) infections five were repeat recruitment of patients during the same clinical episode when referred from Hospital Sarikei to Hospital Sibu ('B' samples). Twenty-four of the remaining patients did not fulfil the study criteria as follows: Three patients were under 15 year, three patients were either pregnant or with a co-morbidity and 17 had received antimalarial treatment prior to recruitment. Therefore 232 patients with single species PCR-confirmed *P. knowlesi* infections fulfilled the study criteria. Of these 161 (69%) *of P. knowlesi* patients were recruited in Hospital Sarikei (including one patient from Kapit) and 71 (31%) in Hospital Sibu. Of the 147 group 99 were recruited in Sarikei and one in Kapit grouped to 100 (68%)and 47 (32%) in Sibu. (**B**) Of 85 patients with non-*P. knowlesi* malaria recruited into the study, six patients with *P. falciparum* malaria had received antimalarial treatment prior to recruitment. Four patients with *P. vivax* had received antimalarial treatment two were pregnant and one had missing laboratory results.
(PDF)

Figure S2 **Differences between men and women with** ***P. knowlesi*** **malaria.** Significant differences in Hemoglobin, Pulse and PVC between men and women were observed when outliers were removed from the data (SPSS and Prism 4 for Macintosh, GraphPad Software, Inc).
(PDF)

Figure S3 **Clinical and laboratory measures of disease progression that associate with** ***P. knowlesi*** **parasitaemia in the unselected patient group (n = 232).** Prism 4 for Macintosh, GraphPad Software, Inc.
(PDF)

Figure S4 **Evidence of** ***Pknbpxa*** **dimorphism.** Neighbor-Joining tree inferred from five *Pknpxa* reference DNA sequences (8501 bp), *P. knowlesi* published sequence EU867191 with *P. falciparum* Rh2a (XM001350047.1) as the out group. The percentage of replicate trees in which the associated taxa clustered together in the bootstrap test (1000 replicates) are shown next to the branches The evolutionary distances were computed using the Jukes-Cantor method. All positions containing gaps and missing data were eliminated. There were a total of 8414 positions in the final dataset. Evolutionary analyses were conducted in MEGA5 (Tamura et al. 2011. Molecular Biology and Evolution, **28** (10) pp2731-2739).
(PDF)

Figure S5 ***Pkbnpxb*** **nucleotide deletions in patient isolates.** Three nucleotide deletions were detected in five *P. knowlesi* reference isolates from patients compared with *P. knowlesi* H-strain *Pknbpxb* published sequence EU867792. Nucleic acid sequences and amino acid translations are shown. The conserved amino acid motif (ENL) in patient isolates and the corresponding GKIY motif in the published *Pknbpxb* amino acid sequence ACJ54536 are shown. Image generated using Geneious 6.0.4
(PDF)

Figure S6 **Differences in markers of disease progression in patients infected with** ***Pknbpxa*** **group 6 alleles.** Significant differences in patents infected with *Pknbpxa* group 6 allele iii 913 C (yellow) compared with the 913T allele (green when in the KH273 dimorphism and blue when in the KH195 dimorphism) are shown. P values were calculated using the unpaired t test except for Serum creatinine. *Serum creatinine levels were not normally distributed and the Mann-Whitney U test was used. Prism 4 for Macintosh, GraphPad Software, Inc. Note that all of the HK273 dimorphic form had the 913T allele.
(PDF)

Figure S7 **Differences in markers of disease progression in patients infected with** ***Pknbpxa*** **group 8 alleles.** Significant differences between patents infected with *Pknbpxa* alleles of the complex non-synonymous polymorphic site 982 (T/G/C). T is the only allele within the KH 273 dimorphism (n-61, green) and this dimorphism clustered with less severe markers of disease progression. The KH195 dimorphism has either 982G (n = 51, blue) or 982C (n = 26, pink) at this position each with some changes in Haemoglobin, Plasma Lactate, AST and IL-10. P values are shown for significant differences and determined using the unpaired t test Prism 4 for Macintosh, GraphPad Software, Inc.
(PDF)

Figure S8 **Differences in markers of disease progression in patents infected with** ***Pknbpxb*** **group 1 alleles (2638 A/C polymorphism).** The 2638A allele ii, n = 20 (green) grouped with low haemoglobin and high axillary temperature. Tests for significant differences between groups: unpaired t test for haemoglobin and the Mann- Whitney U test for axillary temperature, Prism 4 for Macintosh, GraphPad Software, Inc.
(PDF)

Figure S9 **Significant differences in markers of disease progression in patents infected with** ***Pknbpxb*** **group 2 alleles.** Group 2 alleles comprised SNP sites 2117,2740,2757,2802,2834 and 3115 (Table S4). Allele i, n = 51, blue. Allele ii) n = 49, green. Allele iii) n = 31, pink. Unpaired t test for significant between group differences, Prism 4 for Macintosh, GraphPad Software, Inc.
(PDF)

Table S1 **Summary of publications on reticulocyte binding-like proteins in different** ***Plasmodium*** **species.**
(PDF)

Table S2 **Summary of direct PCR sequencing patient isolates for haplotyping.** **a** *Pknbpxa* and **b** *Pknbpxb* gene fragments.
(PDF)

Table S3 **Normality tests for analysis of genetic markers of disease progression.**
(PDF)

Table S4 ***Pknbpxa*** **and** ***Pknbpxb*** **allelic groups and significant differences between alleles in markers of disease severity.**
(PDF)

Table S5 **Reference isolates used for generating full-length (8501 bp)** ***Pknbpxa*** **and (3506 bp)** ***Pknbpxb*** **gene sequences.**
(PDF)

Table S6 ***P. knowlesi*** **polymorphisms in the haplotyping fragment (885 bp) making up the** ***Pknbpxa*** **dimorphism.**
(PDF)

Table S7 **Non-synonymous sites and amino acid changes in** ***P. knowlesi Pknbpxa*** **and** ***Pknbpxb*** **haplotyping fragments.** **a** *Pknbpxa* 138 patient isolates, **b** *Pknbpxb* 134 patient isolates.
(PDF)

Text S1 **Additional information on patient recruitment, blood sample processing and the study cohort.**
(DOCX)
