# Supplementary figures and images for: Disease Progression in Plasmodium knowlesi Malaria Is Linked to Variation in Invasion Gene Family Members
Source: PLoS Negl Trop Dis. 2014 Aug 14;8(8):e3086. doi: 10.1371/journal.pntd.0003086 (PMC4133233; doi:10.1371/journal.pntd.0003086)

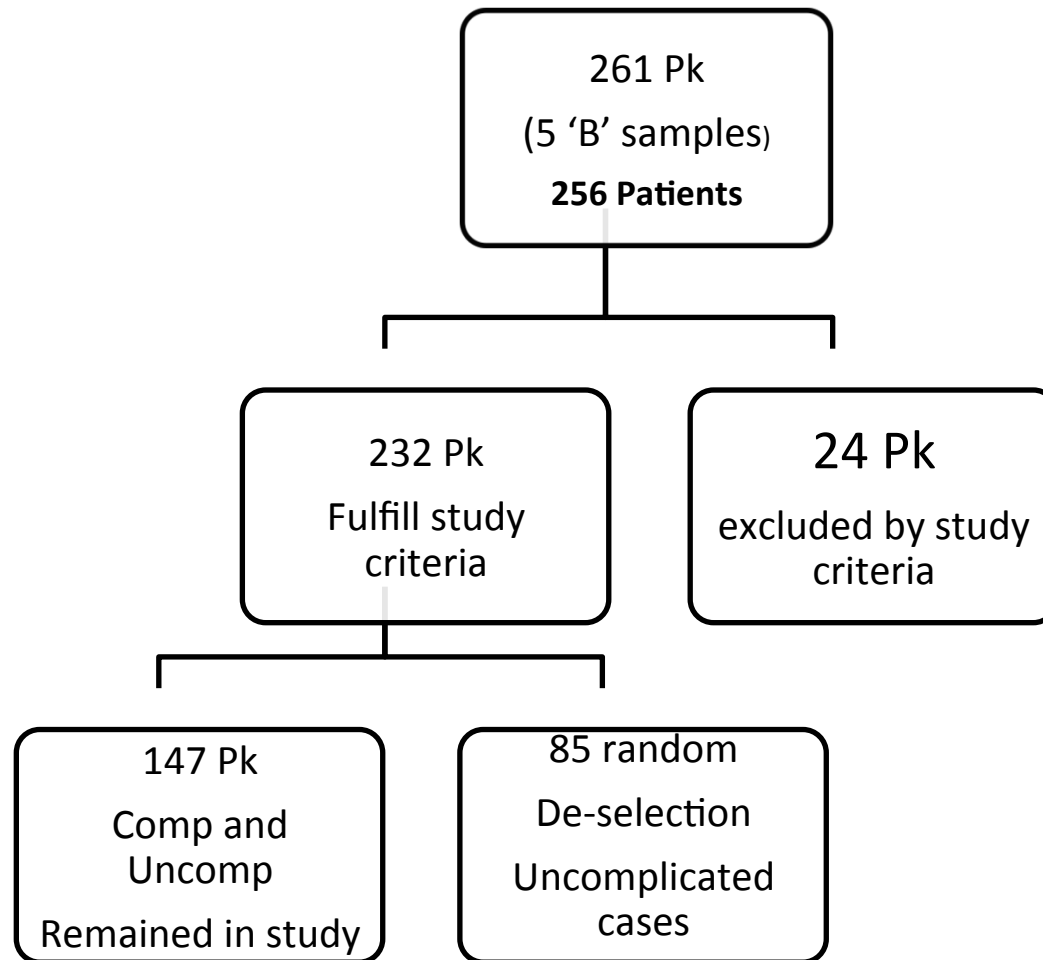

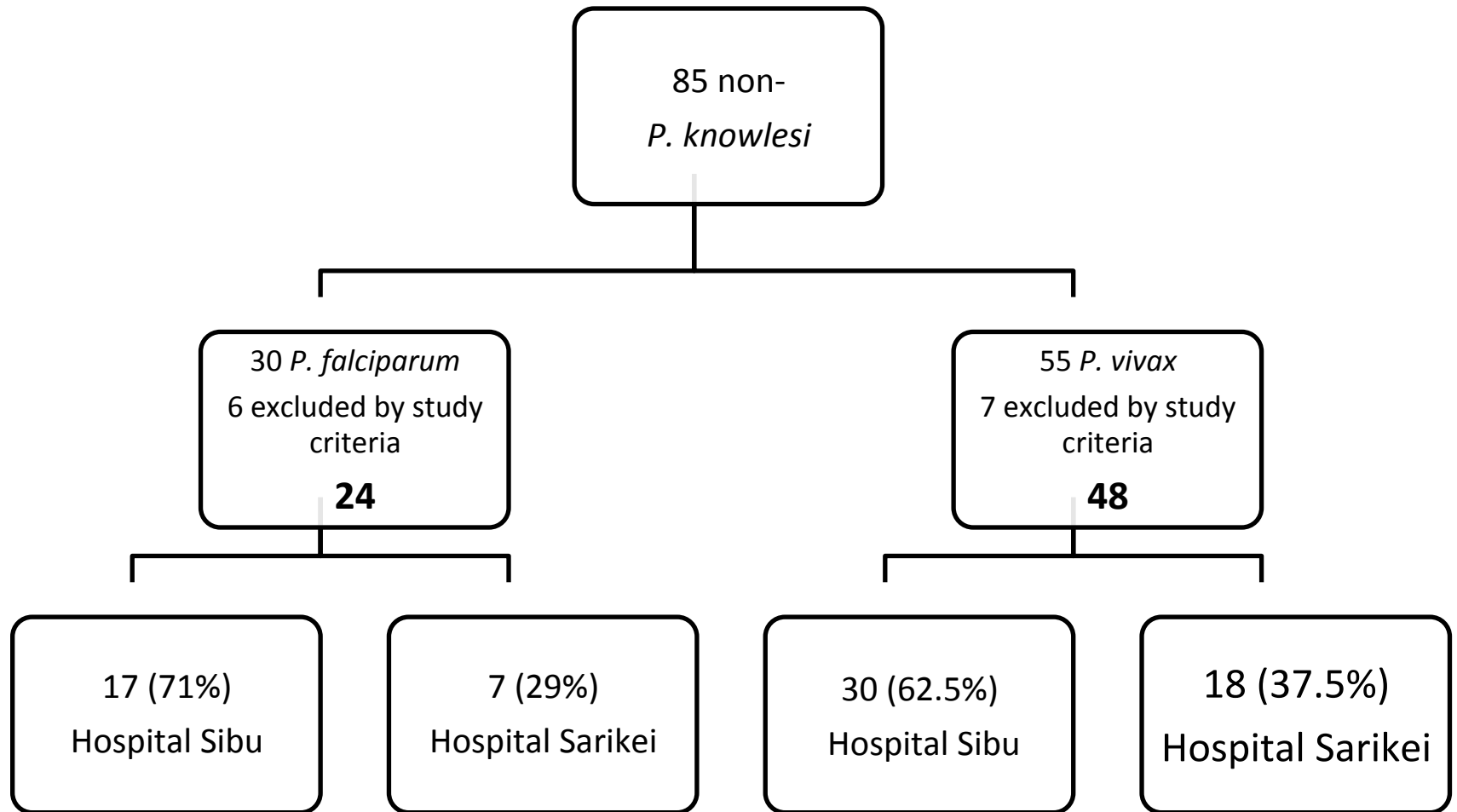

Supplement: Figure S1 — Patients with malaria recruited into the study. (A) Of 261 patients recruited with PCR-confirmed single species P. knowlesi (Pk) infections five were repeat recruitment of patients during the same clinical episode when referred from Hospital Sarikei to Hospital Sibu ('B' samples). Twenty-four of the remaining patients did not fulfil the study criteria as follows: Three patients were under 15 year, three patients were either pregnant or with a co-morbidity and 17 had received antimalarial treatment prior to recruitment. Therefore 232 patients with single species PCR-confirmed P. knowlesi infections fulfilled the study criteria. Of these 161 (69%) of P. knowlesi patients were recruited in Hospital Sarikei (including one patient from Kapit) and 71 (31%) in Hospital Sibu. Of the 147 group 99 were recruited in Sarikei and one in Kapit grouped to 100 (68%)and 47 (32%) in Sibu. (B) Of 85 patients with non-P. knowlesi malaria recruited into the study, six patients with P. falciparum malaria had received antimalarial treatment prior to recruitment. Four patients with P. vivax had received antimalarial treatment two were pregnant and one had missing laboratory results. (PDF) [file pntd.0003086.s001.pdf]

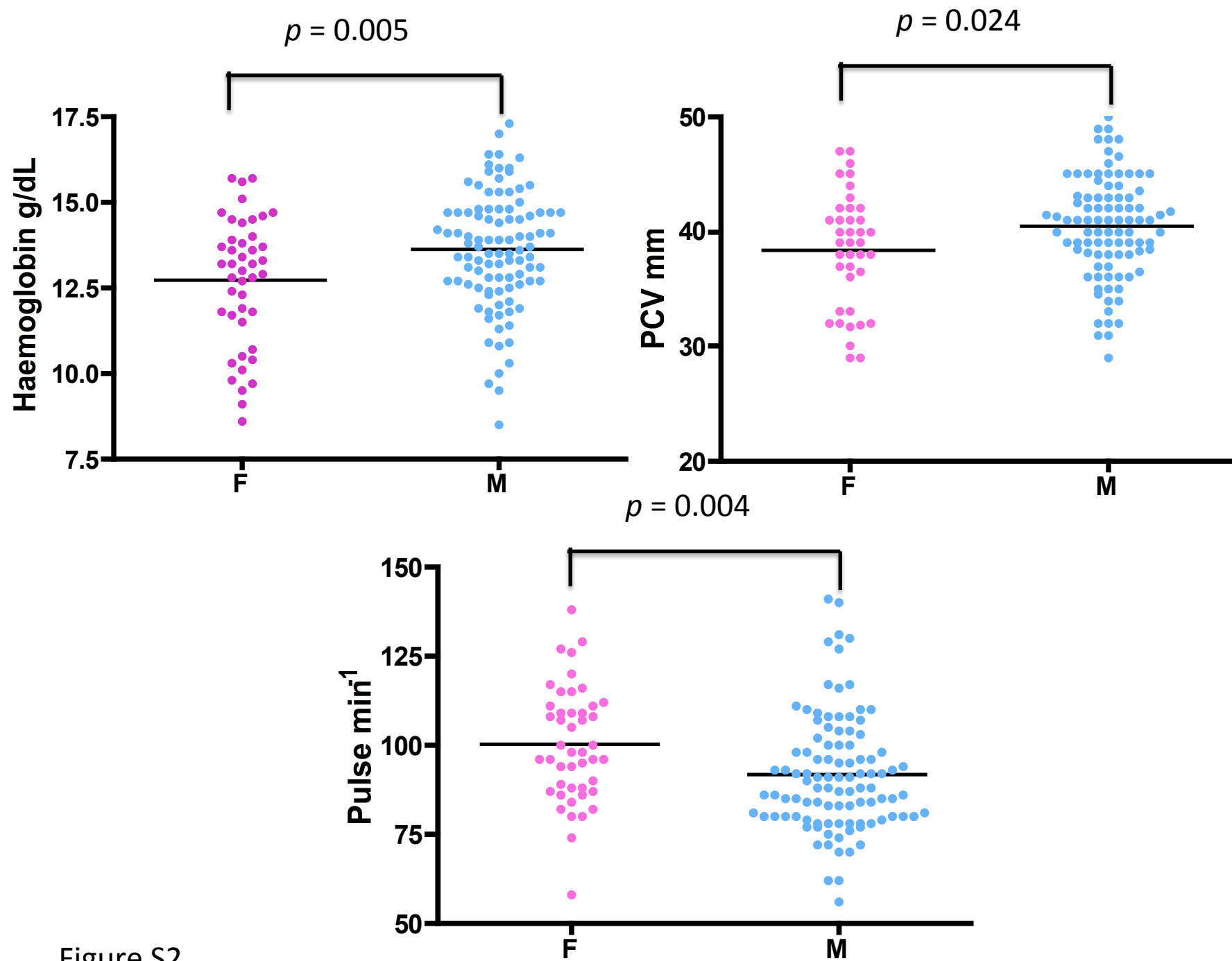

Figure S2

Supplement: Figure S2 — Differences between men and women with P. knowlesi malaria. Significant differences in Hemoglobin, Pulse and PVC between men and women were observed when outliers were removed from the data (SPSS and Prism 4 for Macintosh, GraphPad Software, Inc). (PDF) [file pntd.0003086.s002.pdf]

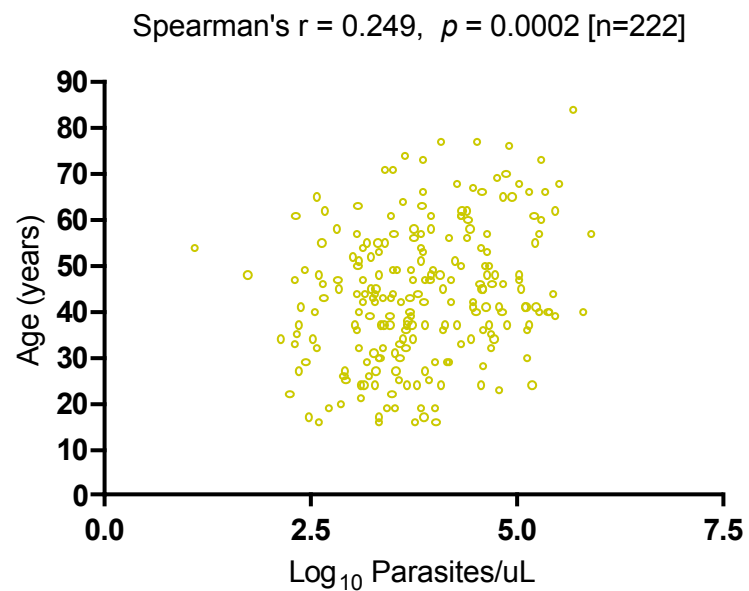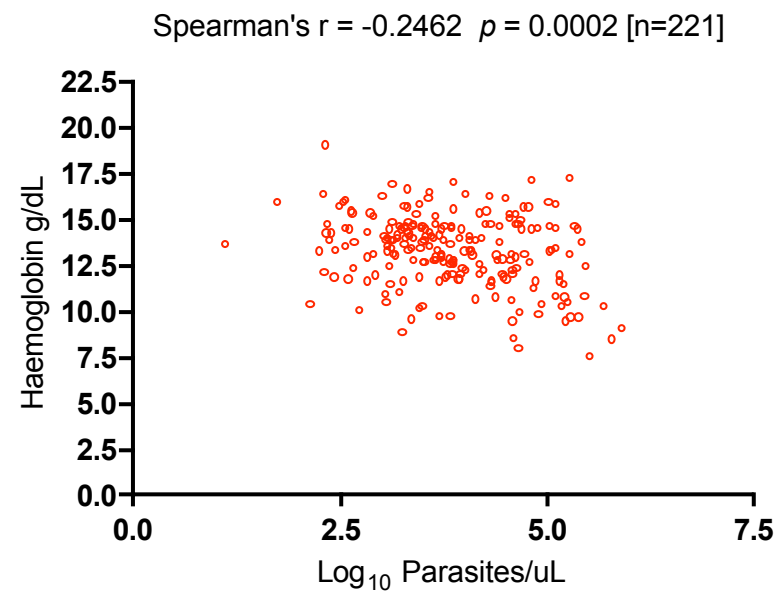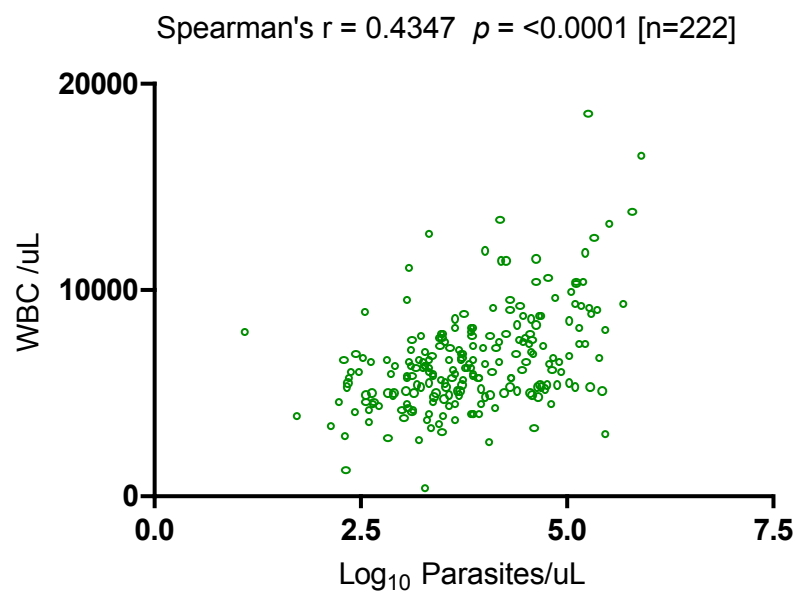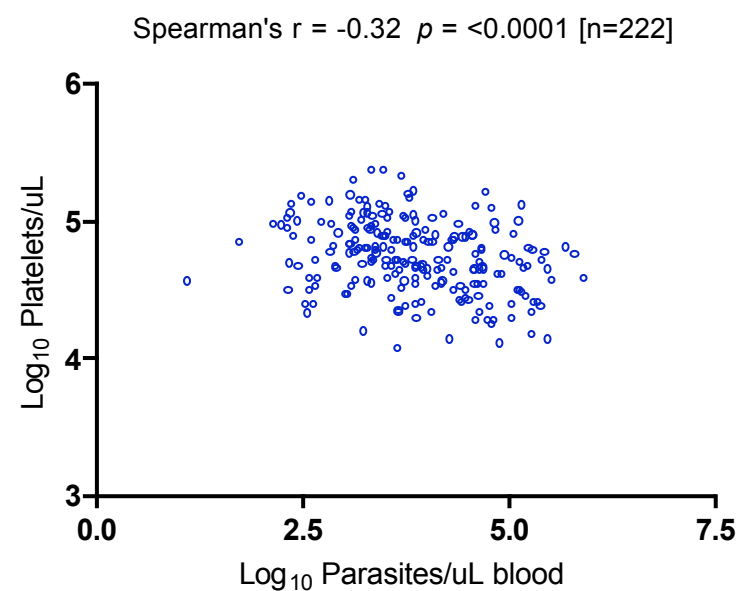

Figure S3

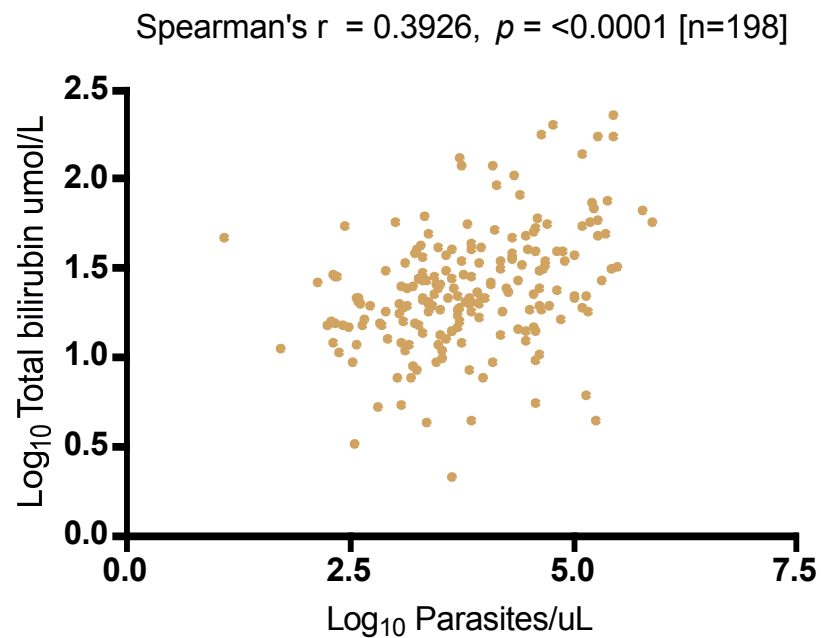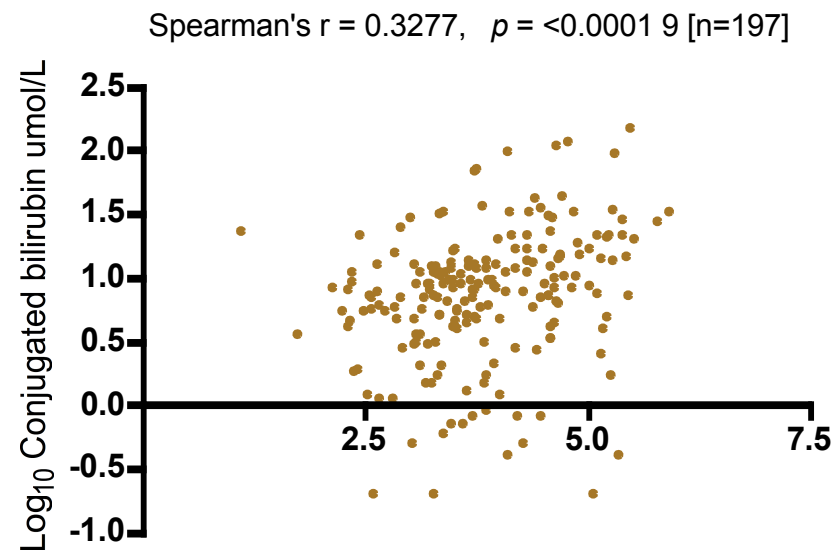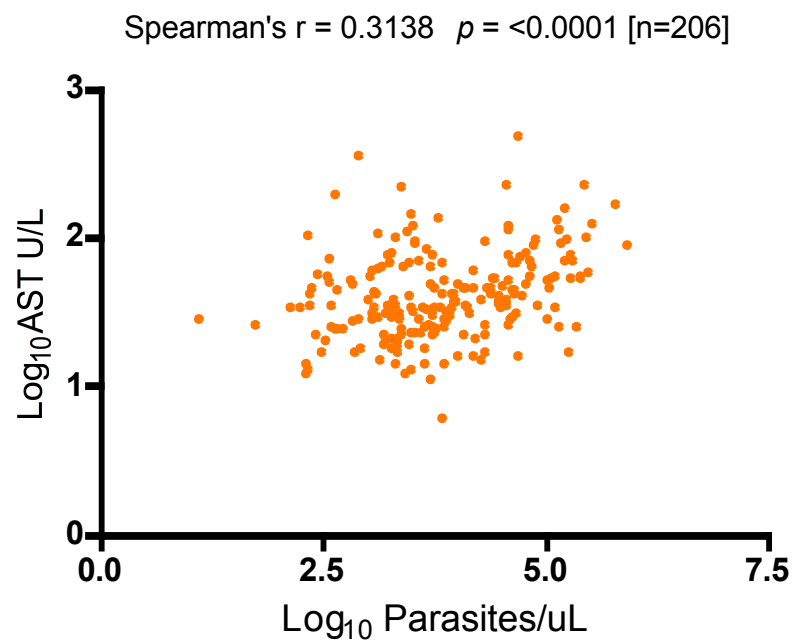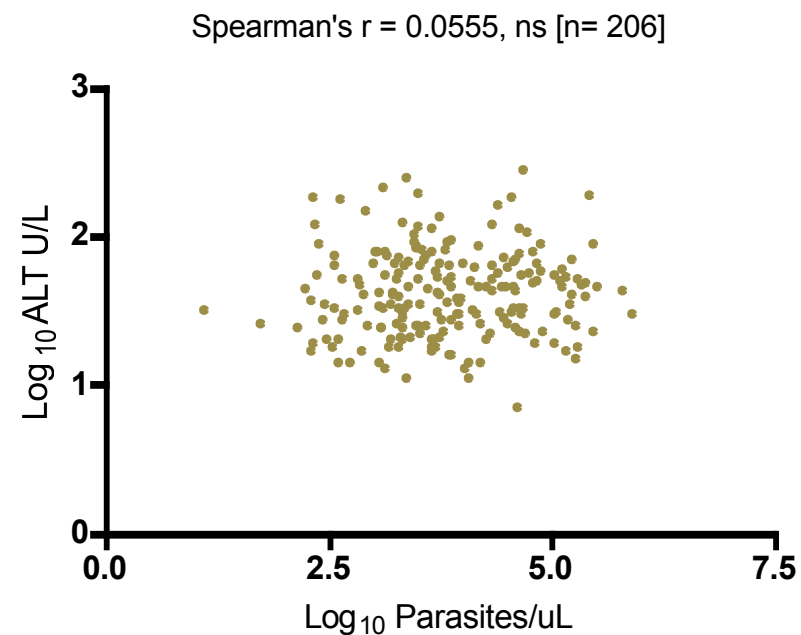

Figure S3

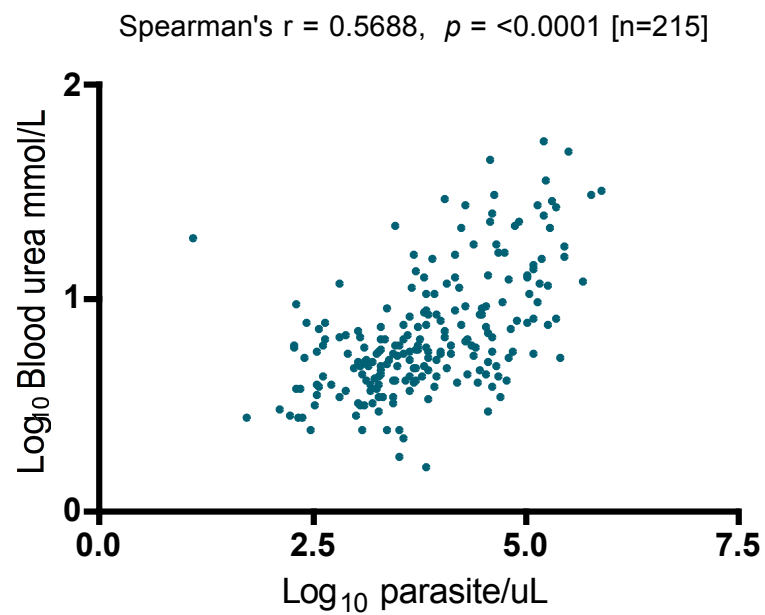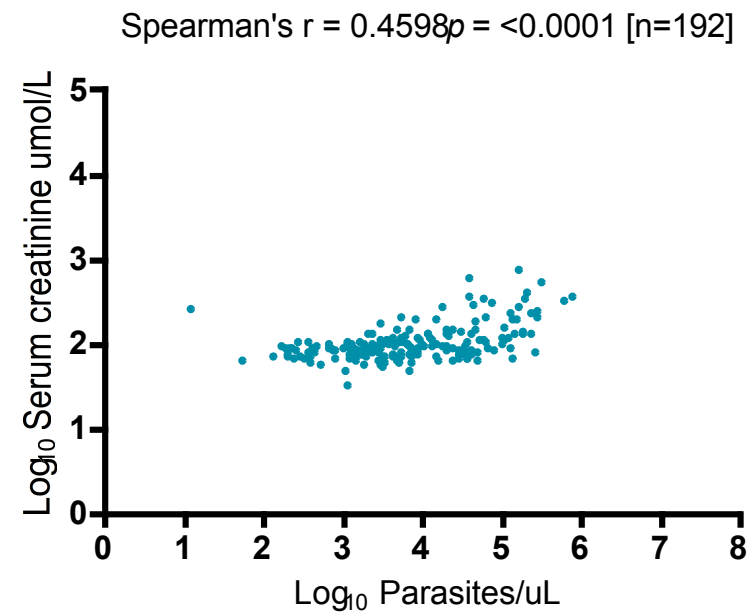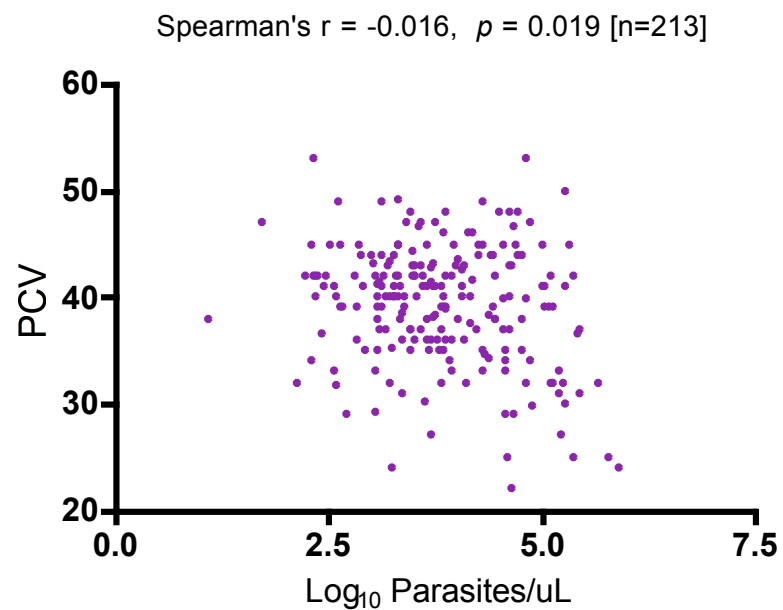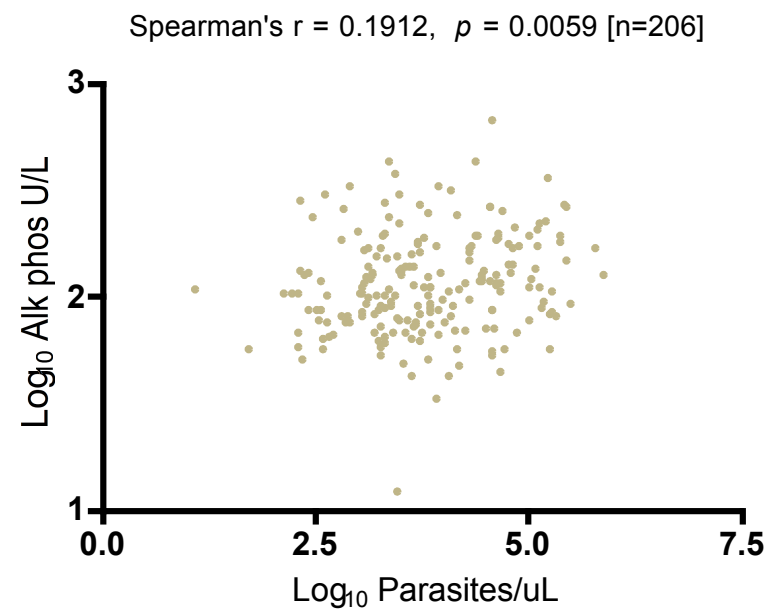

Figure S3

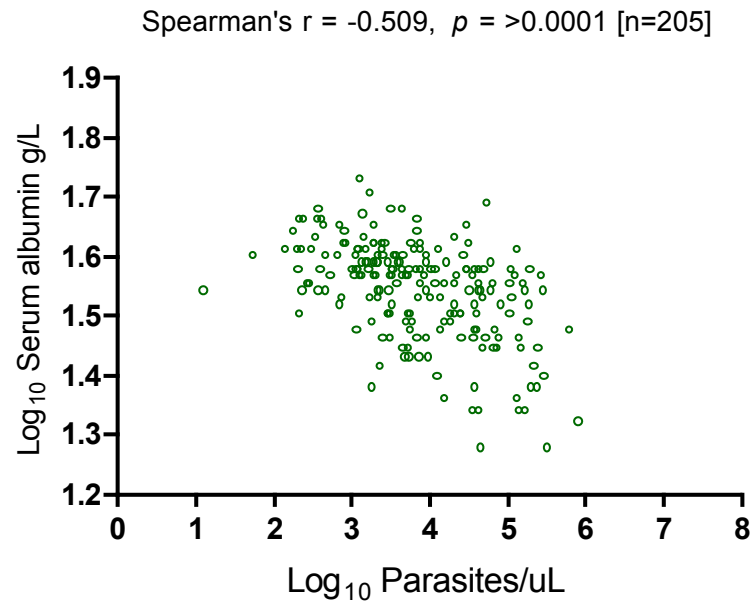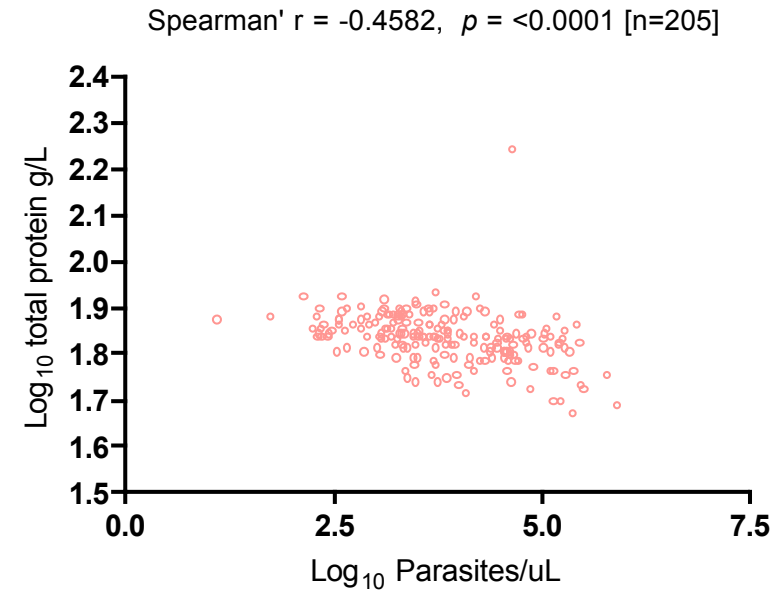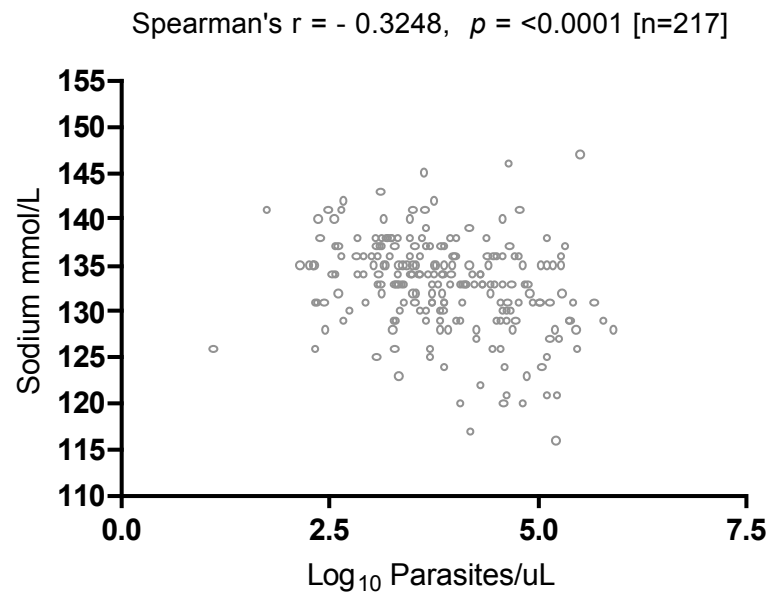

Figure S3

Supplement: Figure S3 — Clinical and laboratory measures of disease progression that associate with P. knowlesi parasitaemia in the unselected patient group (n = 232). Prism 4 for Macintosh, GraphPad Software, Inc. (PDF) [file pntd.0003086.s003.pdf]

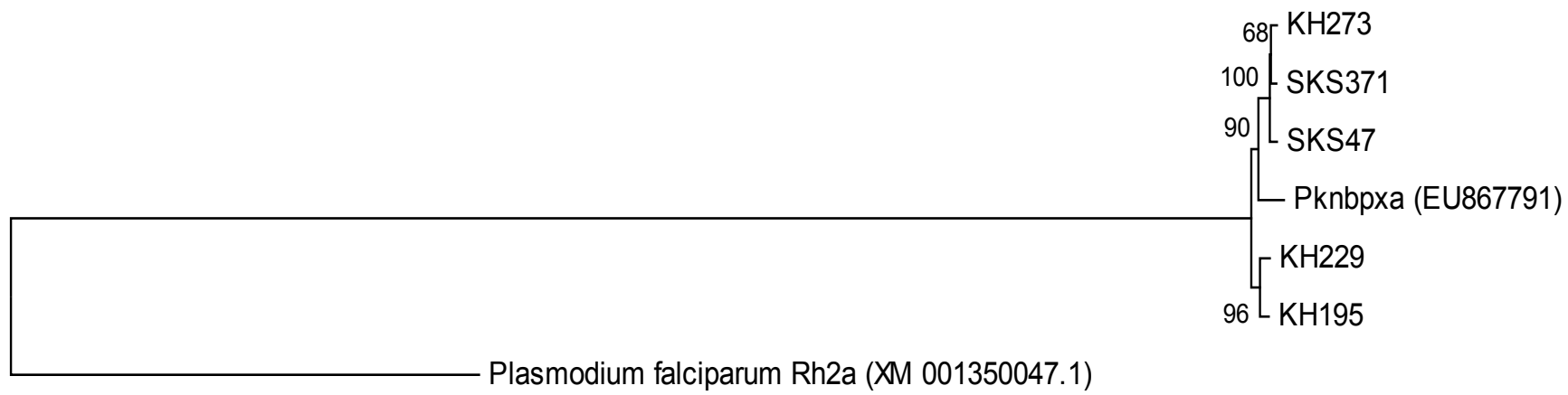

0.01

Figure S4

Supplement: Figure S4 — Evidence of Pknbpxa dimorphism. Neighbor-Joining tree inferred from five Pknpxa reference DNA sequences (8501 bp), P. knowlesi published sequence EU867191 with P. falciparum Rh2a (XM001350047.1) as the out group. The percentage of replicate trees in which the associated taxa clustered together in the bootstrap test (1000 replicates) are shown next to the branches The evolutionary distances were computed using the Jukes-Cantor method. All positions containing gaps and missing data were eliminated. There were a total of 8414 positions in the final dataset. Evolutionary analyses were conducted in MEGA5 (Tamura et al. 2011. Molecular Biology and Evolution, 28 (10) pp2731-2739). (PDF) [file pntd.0003086.s004.pdf]

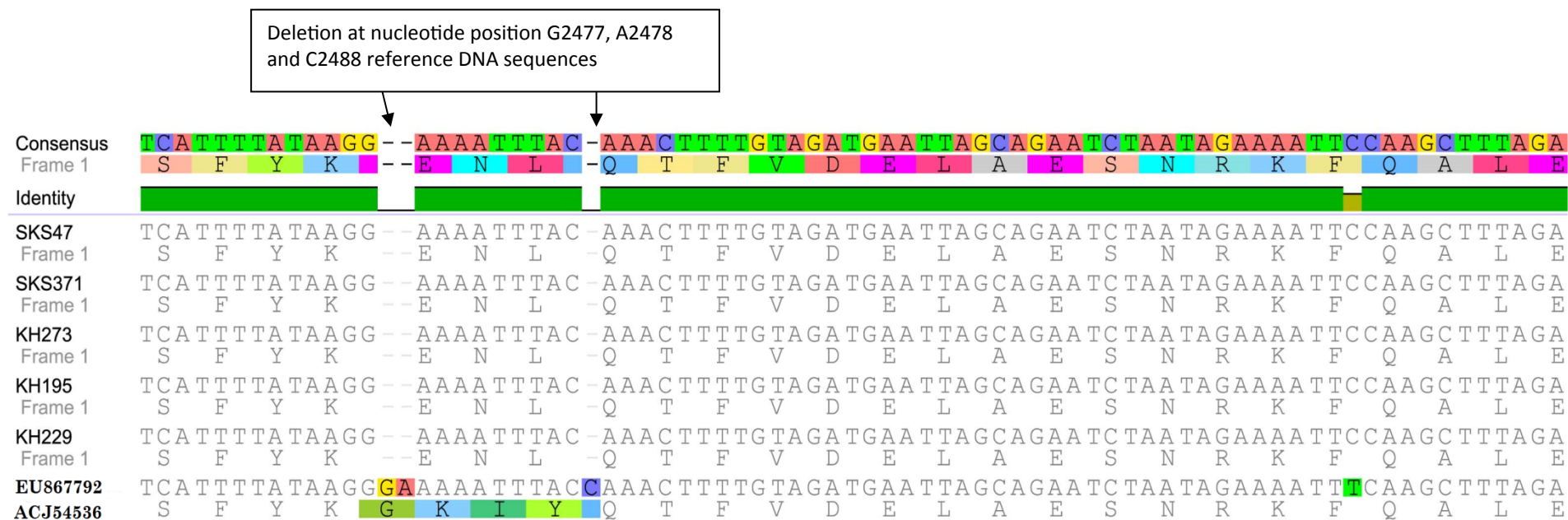

Figure S5

Supplement: Figure S5 — Pkbnpxb nucleotide deletions in patient isolates. Three nucleotide deletions were detected in five P. knowlesi reference isolates from patients compared with P. knowlesi H-strain Pknbpxb published sequence EU867792. Nucleic acid sequences and amino acid translations are shown. The conserved amino acid motif (ENL) in patient isolates and the corresponding GKIY motif in the published Pknbpxb amino acid sequence ACJ54536 are shown. Image generated using Geneious 6.0.4 (PDF) [file pntd.0003086.s005.pdf]

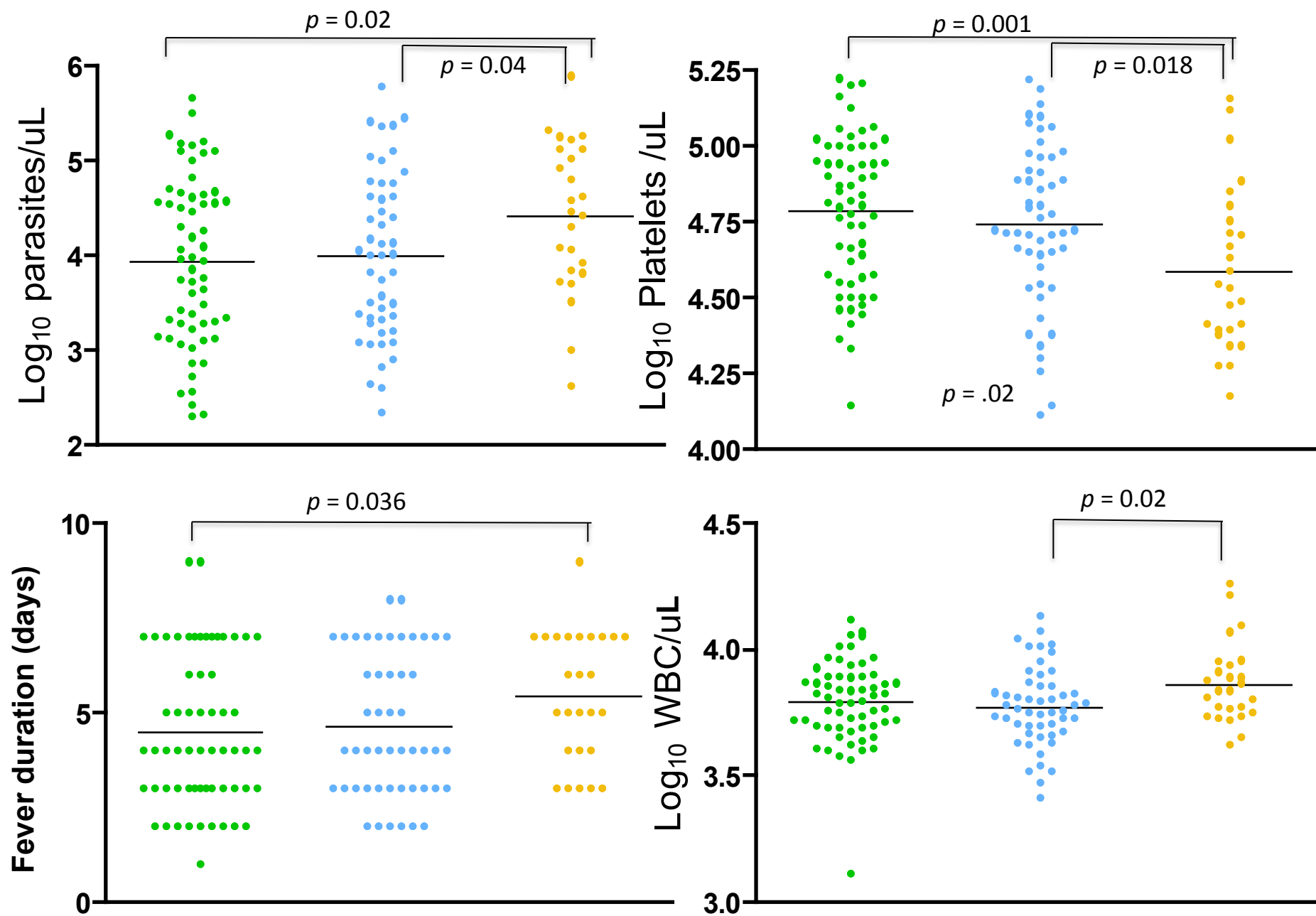

Figure S6

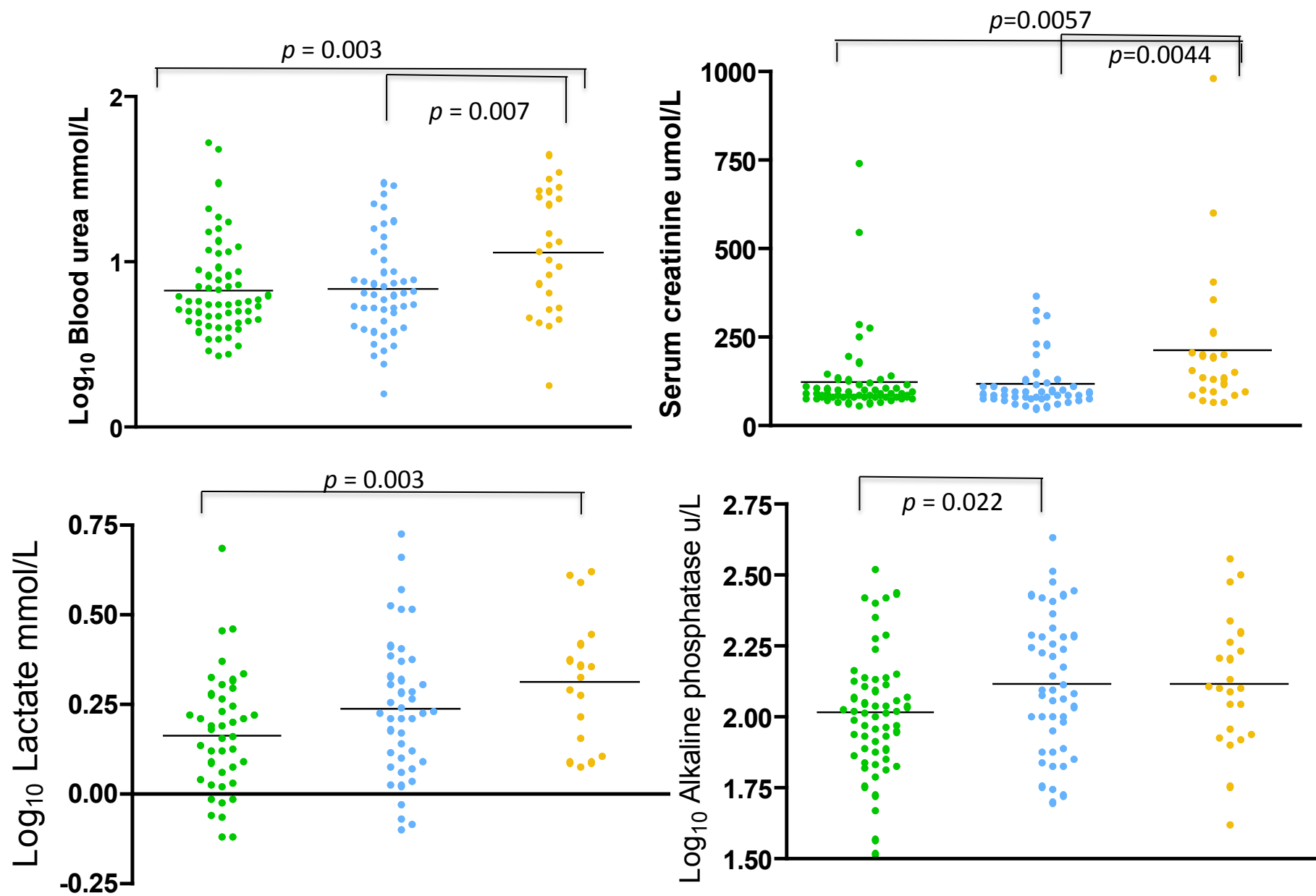

Figure S6

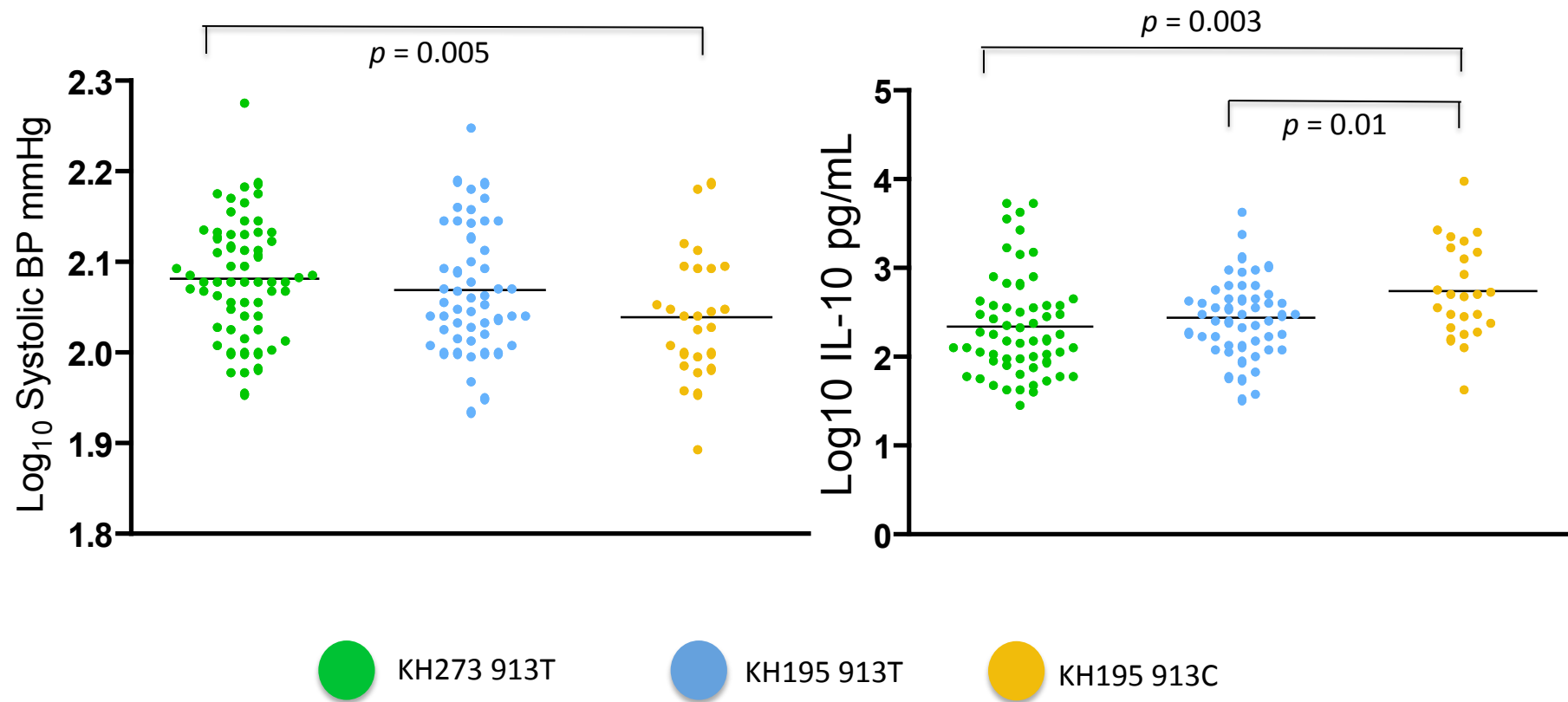

Figure S6

Supplement: Figure S6 — Differences in markers of disease progression in patients infected with Pknbpxa group 6 alleles. Significant differences in patents infected with Pknbpxa group 6 allele iii 913 C (yellow) compared with the 913T allele (green when in the KH273 dimorphism and blue when in the KH195 dimorphism) are shown. P values were calculated using the unpaired t test except for Serum creatinine. *Serum creatinine levels were not normally distributed and the Mann-Whitney U test was used. Prism 4 for Macintosh, GraphPad Software, Inc. Note that all of the HK273 dimorphic form had the 913T allele. (PDF) [file pntd.0003086.s006.pdf]

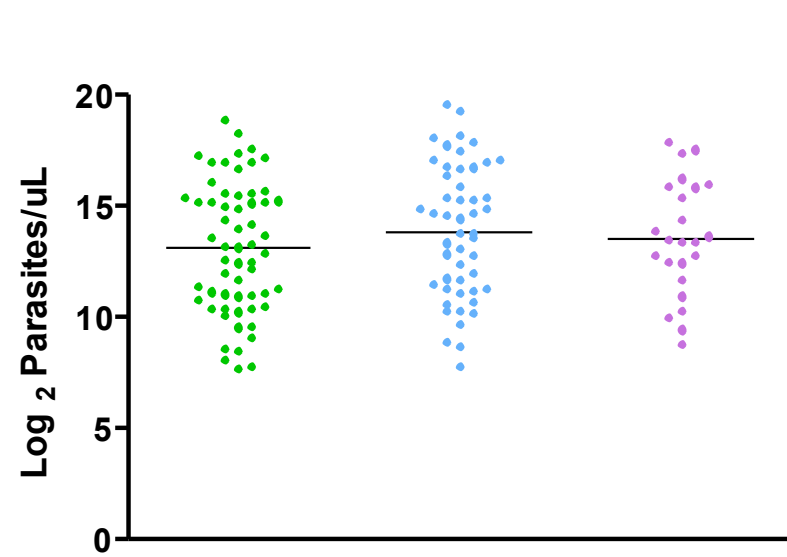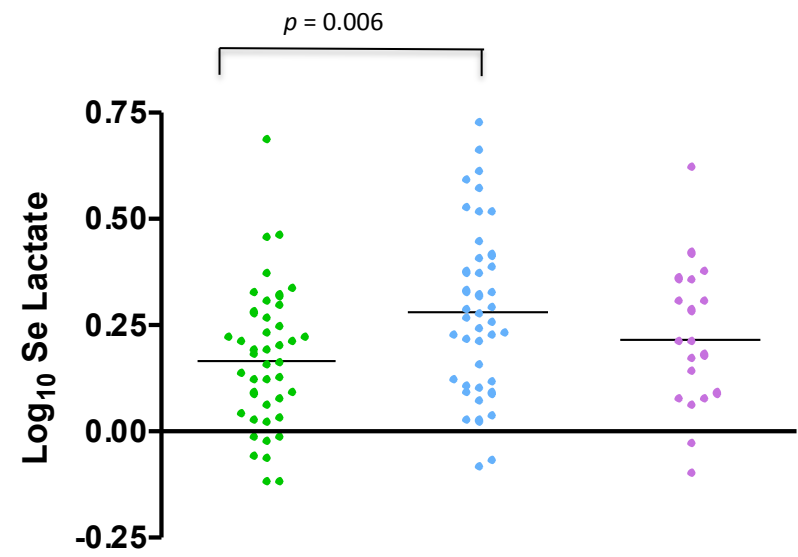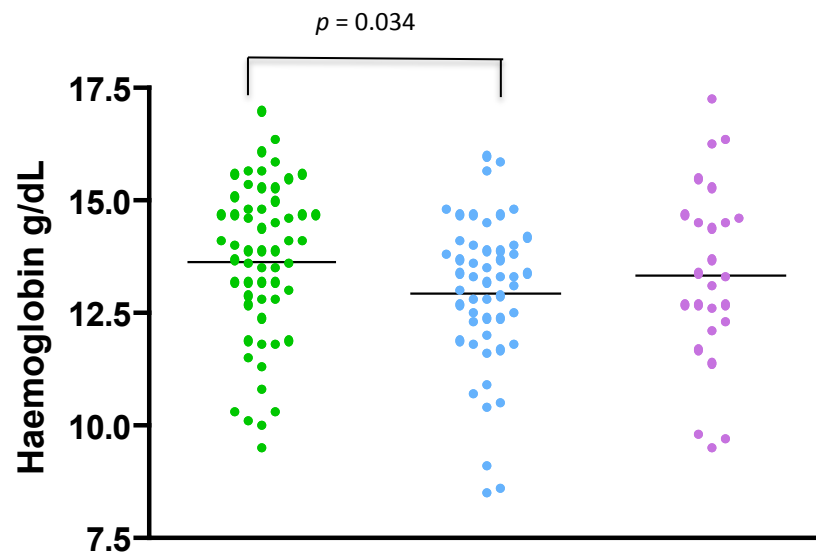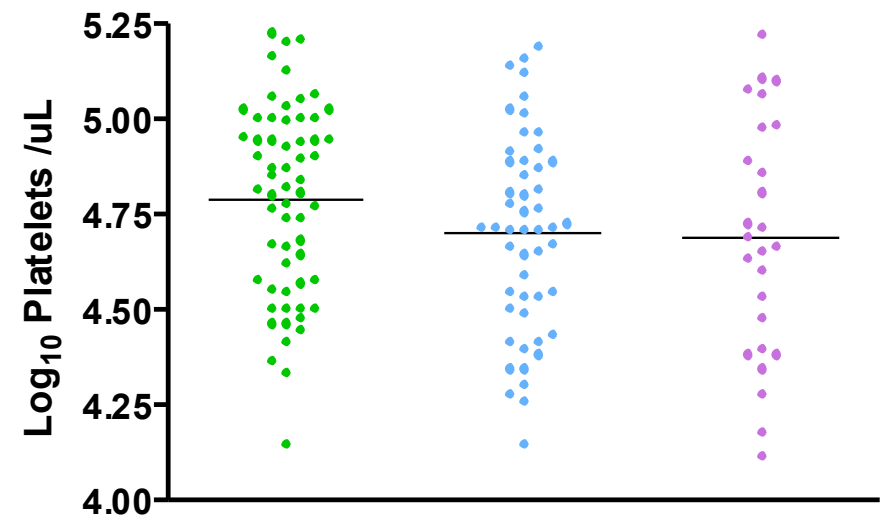

Figure S7

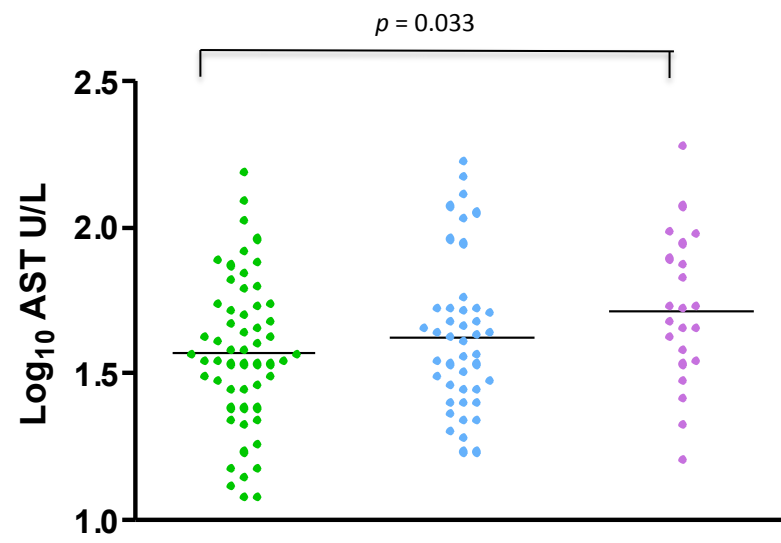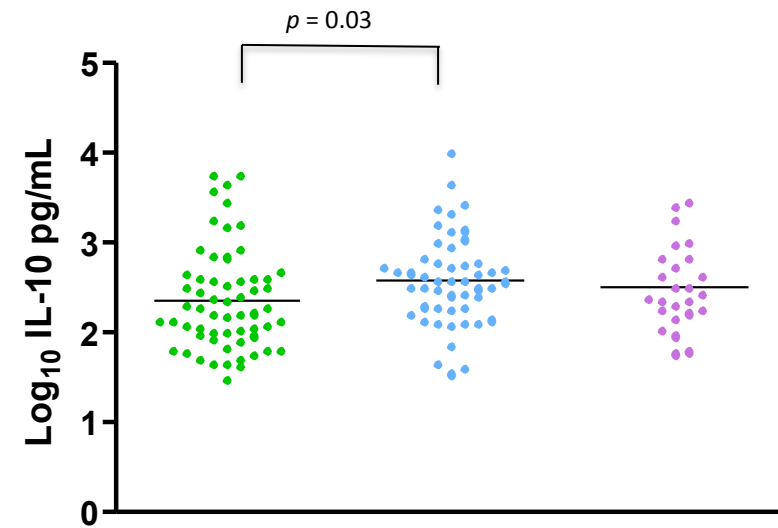

Figure S7

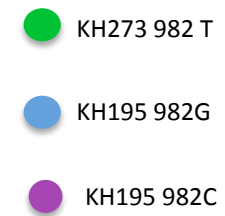

Supplement: Figure S7 — Differences in markers of disease progression in patients infected with Pknbpxa group 8 alleles. Significant differences between patents infected with Pknbpxa alleles of the complex non-synonymous polymorphic site 982 (T/G/C). T is the only allele within the KH 273 dimorphism (n-61, green) and this dimorphism clustered with less severe markers of disease progression. The KH195 dimorphism has either 982G (n = 51, blue) or 982C (n = 26, pink) at this position each with some changes in Haemoglobin, Plasma Lactate, AST and IL-10. P values are shown for significant differences and determined using the unpaired t test Prism 4 for Macintosh, GraphPad Software, Inc. (PDF) [file pntd.0003086.s007.pdf]

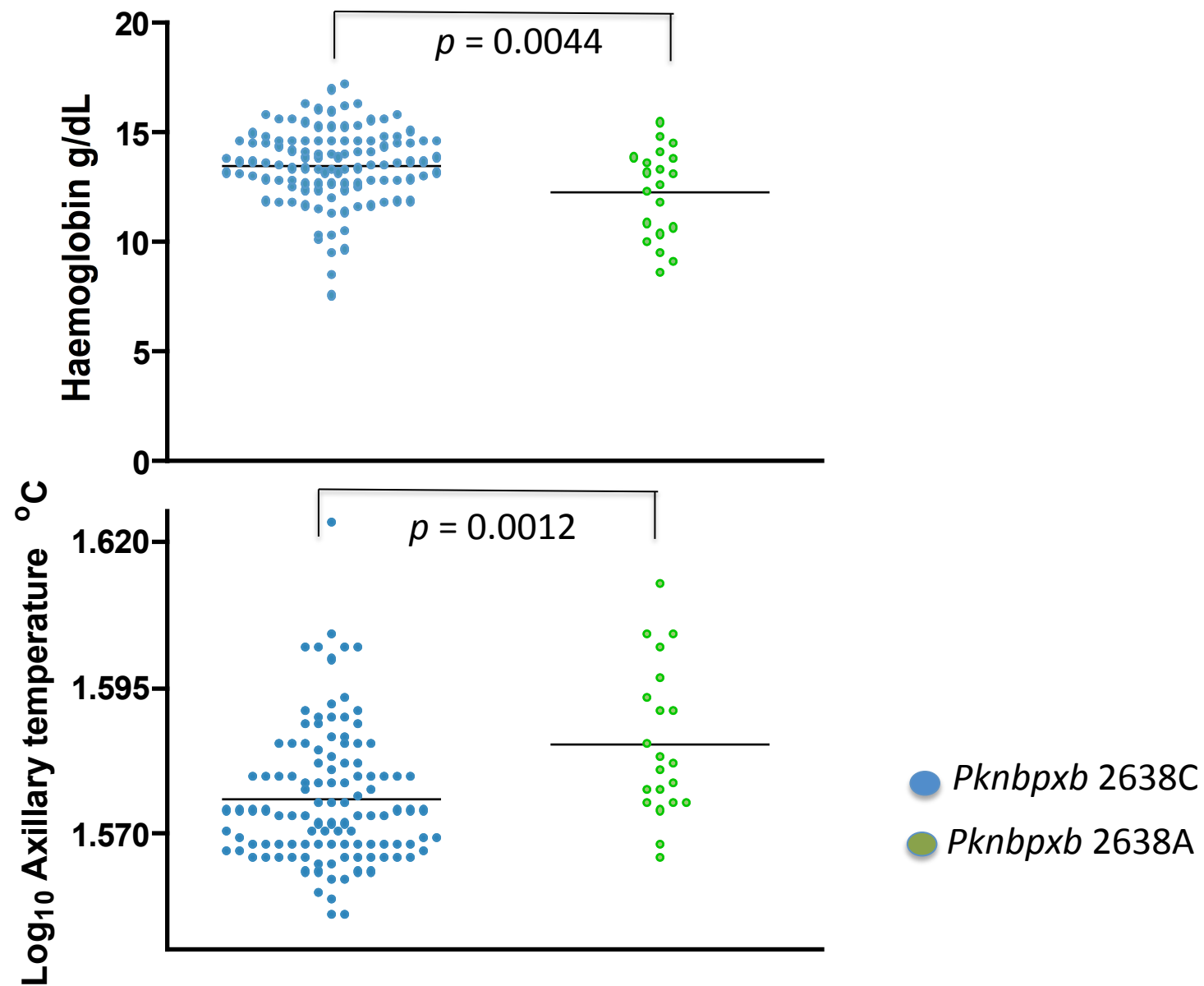

Figure S8

Supplement: Figure S8 — Differences in markers of disease progression in patents infected with Pknbpxb group 1 alleles (2638 A/C polymorphism). The 2638A allele ii, n = 20 (green) grouped with low haemoglobin and high axillary temperature. Tests for significant differences between groups: unpaired t test for haemoglobin and the Mann- Whitney U test for axillary temperature, Prism 4 for Macintosh, GraphPad Software, Inc. (PDF) [file pntd.0003086.s008.pdf]

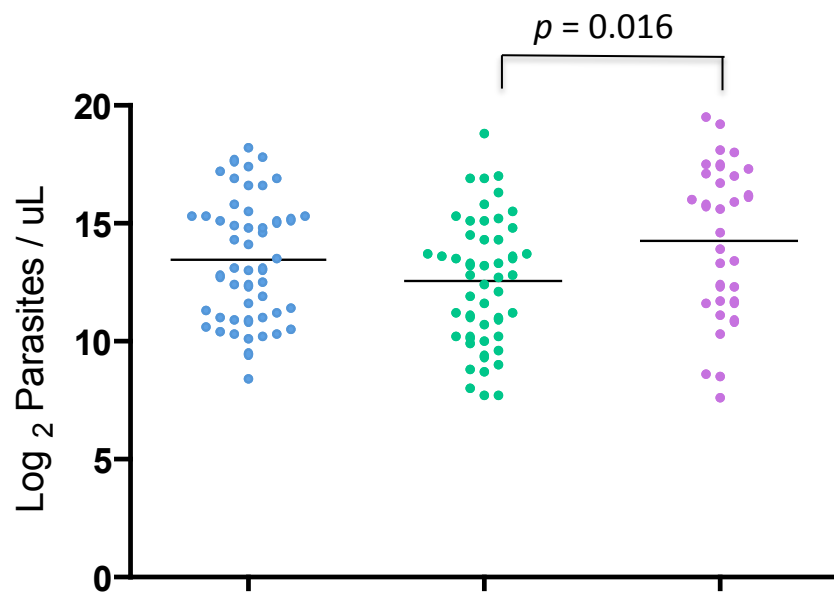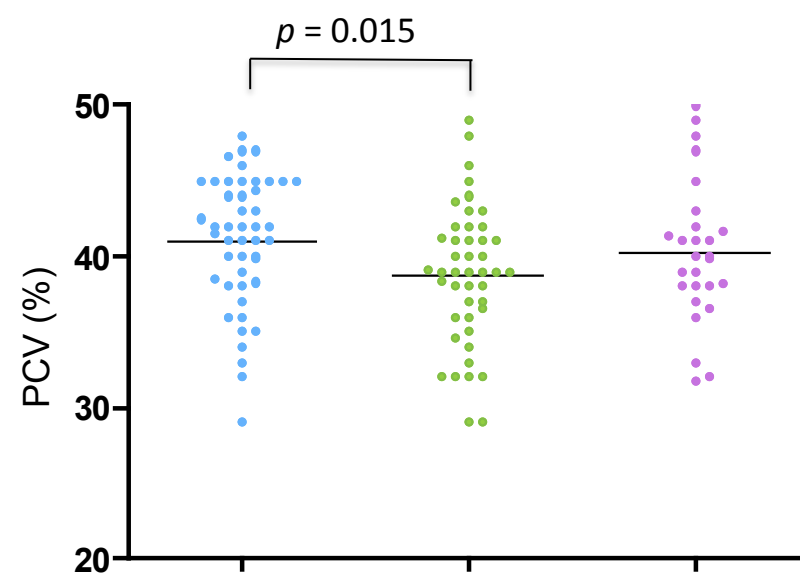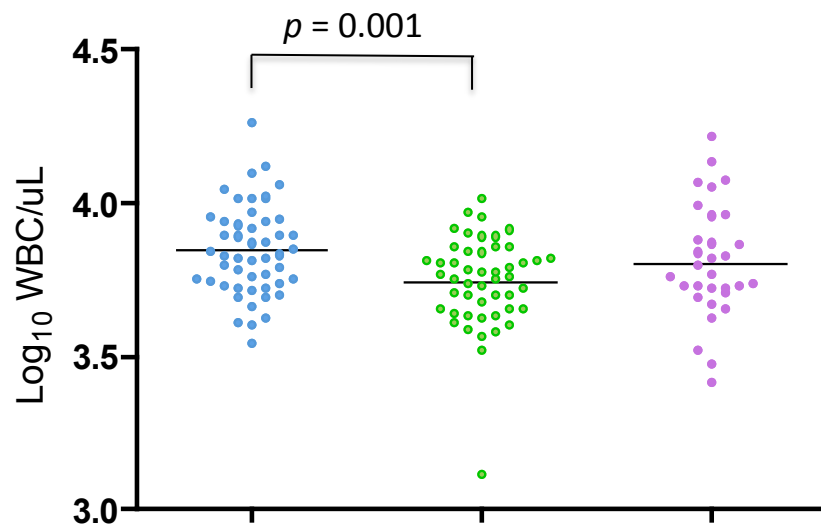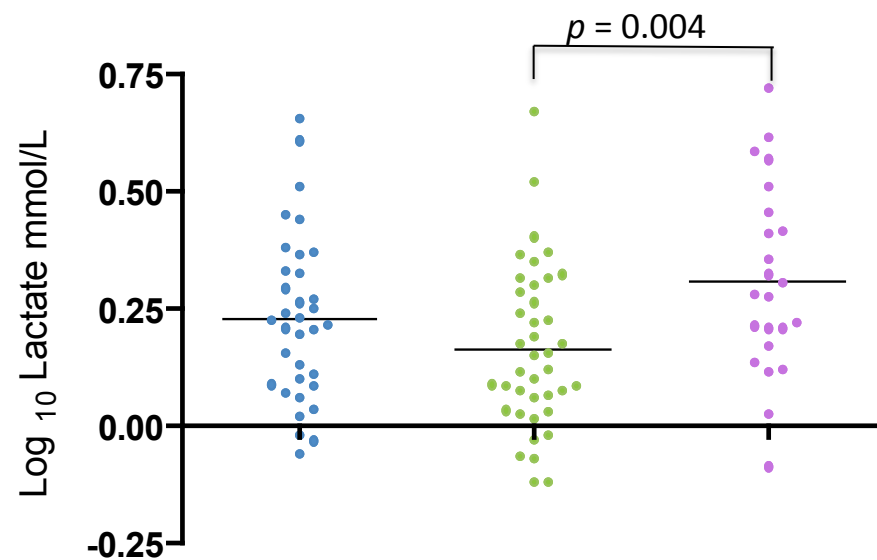

Figure S9

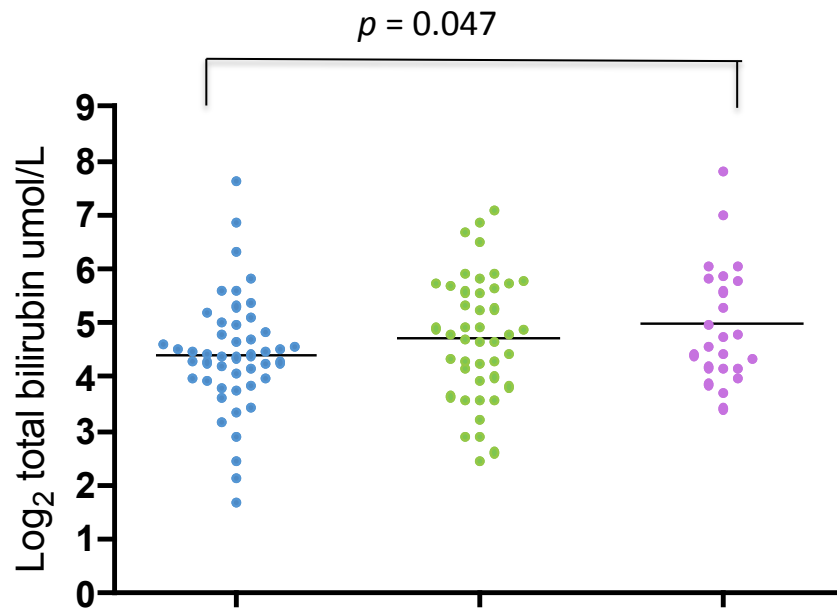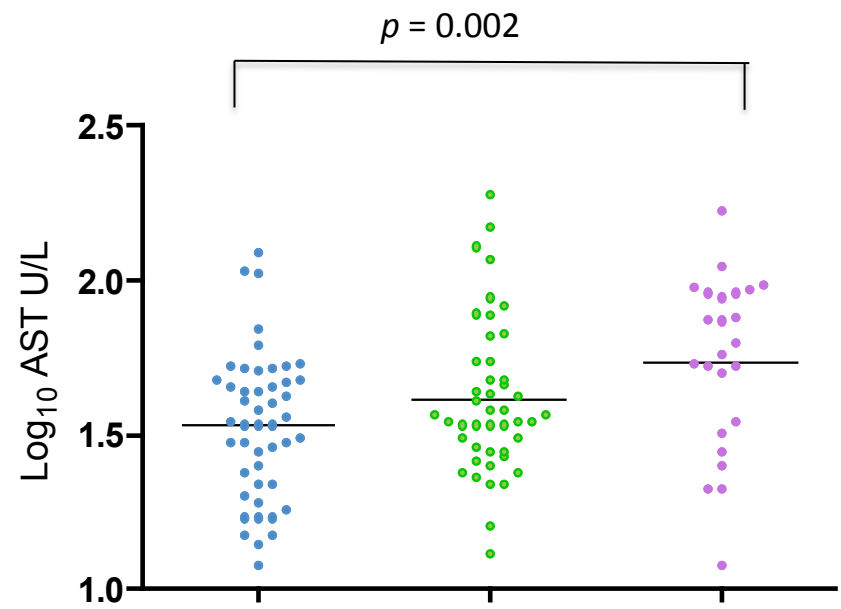

- *Pknbpxb* group 2 allele (i)
- *Pknbpxb* group 2 allele (ii)
- *Pknbpxb* group 2 allele (iii)

Figure S9

Supplement: Figure S9 — Significant differences in markers of disease progression in patents infected with Pknbpxb group 2 alleles. Group 2 alleles comprised SNP sites 2117,2740,2757,2802,2834 and 3115 (Table S4). Allele i, n = 51, blue. Allele ii) n = 49, green. Allele iii) n = 31, pink. Unpaired t test for significant between group differences, Prism 4 for Macintosh, GraphPad Software, Inc. (PDF) [file pntd.0003086.s009.pdf]
