# Supplementary material for: Disease Progression in Plasmodium knowlesi Malaria Is Linked to Variation in Invasion Gene Family Members
Source: PLoS Negl Trop Dis. 2014 Aug 14;8(8):e3086. doi: 10.1371/journal.pntd.0003086 (PMC4133233; doi:10.1371/journal.pntd.0003086)
Supplement: Table S1 — Summary of publications on reticulocyte binding-like proteins in different Plasmodium species. (PDF) [file pntd.0003086.s010.pdf]

Table S1 Summary of publications on Reticulocyte Binding Proteins in different Plasmodium species.

Species	Protein/Gene	Reference
P. falciparum	Reticulocyte binding like protein homologue (Rh) – PfRh1/PfRh2a/PfRh2b/PfRh4/PfRh5	[1]; [2]; [3]; [4];.[5]; [6]; [7]; [8]; [9]; [10]; [11];. [12]; [13]; [14]; [15]; [16]; [17]; [18]; [19]; [20]; [21]; [22]; [23]; [24]; [25]; [26]
P. vivax	Reticulocyte binding protein (RBP) – PvRBP1/PvRBP2	[27]; [28]; [29]; [30]; [31];
P. knowlesi	Normocyte binding protein (NBP) – PkNBPXa/PkNBPXb	[1]; [31]; [32]
P. cynomolgi	Reticulocyte binding like (RBP)	[1]; [32]; [33]; [34]
P. y. yoelii	Py235 family	[1]; [35]; [36]; [36]; [36]; [39]; [40]

1. Semenya AA, Tran TM, Meyer EV, Barnwell JW, Galinski MR (2012) Two functional reticulocyte binding-like (RBL) invasion ligands of zoonotic Plasmodium knowlesi exhibit differential adhesion to monkey and human erythrocytes. Malar J 11: 228.

2. Lopaticki S, Maier AG, Thompson J, Wilson DW, Tham WH, et al. (2011) Reticulocyte and erythrocyte binding-like proteins function cooperatively in invasion of human erythrocytes by malaria parasites. Infect Immun 79: 1107-1117.
3. Tham WH, Healer J, Cowman AF (2012) Erythrocyte and reticulocyte binding-like proteins of Plasmodium falciparum. Trends Parasitol 28: 23-30.
4. Triglia T, Tham WH, Hodder A, Cowman AF (2009) Reticulocyte binding protein homologues are key adhesins during erythrocyte invasion by Plasmodium falciparum. Cell Microbiol 11: 1671-1687.
5. Triglia T, Chen L, Lopaticki S, Dekiwadia C, Riglar DT, et al. (2011) Plasmodium falciparum merozoite invasion is inhibited by antibodies that target the PfRh2a and b binding domains. PLoS Pathog 7: e1002075.
6. Gunalan K, Gao X, Liew KJ, Preiser PR (2011) Differences in erythrocyte receptor specificity of different parts of the Plasmodium falciparum reticulocyte binding protein homologue 2a. Infect Immun 79: 3421-3430.
7. Gao X, Yeo KP, Aw SS, Kuss C, Iyer JK, et al. (2008) Antibodies targeting the PfRH1 binding domain inhibit invasion of Plasmodium falciparum merozoites. PLoS Pathog 4: e1000104.
8. Sahar T, Reddy KS, Bharadwaj M, Pandey AK, Singh S, et al. (2011) Plasmodium falciparum reticulocyte binding-like homologue protein 2

(PfRH2) is a key adhesive molecule involved in erythrocyte invasion. PLoS One 6: e17102.
9. Rayner JC (2008) The merozoite has landed: reticulocyte-binding-like ligands and the specificity of erythrocyte recognition. Trends Parasitol 25: 104-106.
10. Kaneko O, Mu J, Tsuboi T, Su X, Torii M (2002) Gene structure and expression of a Plasmodium falciparum 220-kDa protein homologous to the Plasmodium vivax reticulocyte binding proteins. Mol Biochem Parasitol 121: 275-278.
11. Rayner JC, Galinski MR, Ingravallo P, Barnwell JW (2000) Two Plasmodium falciparum genes express merozoite proteins that are related to Plasmodium vivax and Plasmodium yoelii adhesive proteins involved in host cell selection and invasion. Proc Natl Acad Sci U S A 97: 9648-9653.
12. Rayner JC, Vargas-Serrato E, Huber CS, Galinski MR, Barnwell JW (2001) A Plasmodium falciparum homologue of Plasmodium vivax reticulocyte binding protein (PvRBP1) defines a trypsin-resistant erythrocyte invasion pathway. J Exp Med 194: 1571-1581.
13. Dvorin JD, Bei AK, Coleman BI, Duraisingh MT (2010) Functional diversification between two related Plasmodium falciparum merozoite invasion ligands is determined by changes in the cytoplasmic domain. Mol Microbiol 75: 990-1006.
14. DeSimone TM, Bei AK, Jennings CV, Duraisingh MT (2009) Genetic analysis of the cytoplasmic domain of the PfRh2b merozoite invasion protein of Plasmodium falciparum. Int J Parasitol 39: 399-405.
15. Ahouidi AD, Bei AK, Neafsey DE, Sarr O, Volkman S, et al. (2010) Population genetic analysis of large sequence polymorphisms in Plasmodium falciparum blood-stage antigens. Infect Genet Evol 10: 200-206.
16. Gaur D, Singh S, Singh S, Jiang L, Diouf A, et al. (2007) Recombinant Plasmodium falciparum reticulocyte homology protein 4 binds to erythrocytes and blocks invasion. Proc Natl Acad Sci U S A 104: 17789-17794.
17. Tham WH, Wilson DW, Lopaticki S, Schmidt CQ, Tetteh-Quarcoo PB, et al. (2010) Complement receptor 1 is the host erythrocyte receptor for Plasmodium falciparum PfRh4 invasion ligand. Proc Natl Acad Sci U S A 107: 17327-17332.
18. Stubbs J, Simpson KM, Triglia T, Plouffe D, Tonkin CJ, et al. (2005) Molecular mechanism for switching of P. falciparum invasion pathways into human erythrocytes. Science 309: 1384-1387.
19. Rodriguez M, Lustigman S, Montero E, Oksov Y, Lobo CA (2008) PfRH5: a novel reticulocyte-binding family homolog of plasmodium falciparum that binds to the erythrocyte, and an investigation of its receptor. PLoS One 3: e3300.
20. Cowman AF, Crabb BS (2006) Invasion of red blood cells by malaria parasites. Cell 124: 755-766.
21. Miller LH, Ackerman HC, Su XZ, Wellems TE (2013) Malaria biology and disease pathogenesis: insights for new treatments. Nat Med 19: 156-167.
22. Crosnier C, Bustamante LY, Bartholdson SJ, Bei AK, Theron M, et al. (2011) Basigin is a receptor essential for erythrocyte invasion by Plasmodium falciparum. Nature 480: 534-537.

23. Chen L, Lopaticki S, Riglar DT, Dekiwadia C, Uboldi AD, et al. (2011) An EGF-like protein forms a complex with PfRh5 and is required for invasion of human erythrocytes by Plasmodium falciparum. PLoS Pathog 7: e1002199.
24. Papadopoulos K (2012) Targeting PfRh5 on Merozoites to Prevent Basigin Binding. Malaria Journal 11: P124.
25. Hayton K, Gaur D, Liu A, Takahashi J, Henschen B, et al. (2008) Erythrocyte binding protein PfRH5 polymorphisms determine species-specific pathways of Plasmodium falciparum invasion. Cell Host Microbe 4: 40-51.
26. Bustamante LY, Bartholdson SJ, Crosnier C, Campos MG, Wanaguru M, et al. (2013) A full-length recombinant Plasmodium falciparum PfRH5 protein induces inhibitory antibodies that are effective across common PfRH5 genetic variants. Vaccine 31: 373-379.
27. Galinski MR, Medina CC, Ingravallo P, Barnwell JW (1992) A reticulocyte-binding protein complex of Plasmodium vivax merozoites. Cell 69: 1213-1226.
28. Galinski MR, Xu M, Barnwell JW (2000) Plasmodium vivax reticulocyte binding protein-2 (PvRBP-2) shares structural features with PvRBP-1 and the Plasmodium yoelii 235 kDa rhoptry protein family. Molecular and biochemical parasitology 108: 257-262.
29. Kosaisavee V, Lek-Uthai U, Suwanarusk R, Gruner AC, Russell B, et al. (2012) Genetic diversity in new members of the reticulocyte binding protein family in Thai Plasmodium vivax isolates. PLoS One 7: e32105.
30. Li J, Han ET (2012) Dissection of the Plasmodium vivax reticulocyte binding-like proteins (PvRBPs). Biochem Biophys Res Commun 426: 1-6.
31. Meyer EV, Semenya AA, Okenu DM, Dluzewski AR, Bannister LH, et al. (2009) The reticulocyte binding-like proteins of P. knowlesi locate to the micronemes of merozoites and define two new members of this invasion ligand family. Mol Biochem Parasitol 165: 111-121.
32. Tachibana S, Sullivan SA, Kawai S, Nakamura S, Kim HR, et al. (2012) Plasmodium cynomolgi genome sequences provide insight into Plasmodium vivax and the monkey malaria clade. Nat Genet 44: 1051-1055.
33. Gunalan K, Gao X, Yap SS, Huang X, Preiser PR (2013) The role of the reticulocyte-binding-like protein homologues of Plasmodium in erythrocyte sensing and invasion. Cell Microbiol 15: 35-44.
34. Okenu DM, Meyer EV, Puckett TC, Rosas-Acosta G, Barnwell JW, et al. (2005) The reticulocyte binding proteins of Plasmodium cynomolgi: a model system for studies of P. vivax. Mol Biochem Parasitol 143: 116-120.
35. Carlton JM, Angiuoli SV, Suh BB, Kooij TW, Pertea M, et al. (2002) Genome sequence and comparative analysis of the model rodent malaria parasite Plasmodium yoelii yoelii. Nature 419: 512-519.
36. Holder AA, Freeman RR (1981) Immunization against blood-stage rodent malaria using purified parasite antigens. Nature 294: 361-364.
37. Freeman RR, Trejdosiewicz AJ, Cross GA (1980) Protective monoclonal antibodies recognising stage-specific merozoite antigens of a rodent malaria parasite. Nature 284: 366-368.

38. Gruber A, Gunalan K, Ramalingam JK, Manimekalai MS, Gruber G, et al. (2011) Structural characterization of the erythrocyte binding domain of the reticulocyte binding protein homologue family of Plasmodium yoelii. Infect Immun 79: 2880-2888.
39. Rayner JC, Huber CS, Galinski MR, Barnwell JW (2004) Rapid evolution of an erythrocyte invasion gene family: the Plasmodium reichenowi Reticulocyte Binding Like (RBL) genes. Molecular and Biochemical Parasitology 133: 287-296.
40. Gruner AC, Snounou G, Fuller K, Jarra W, Renia L, et al. (2004) The Py235 proteins: glimpses into the versatility of a malaria multigene family. Microbes Infect 6: 864-873.
