# Supplementary material for: Disease Progression in Plasmodium knowlesi Malaria Is Linked to Variation in Invasion Gene Family Members
Source: PLoS Negl Trop Dis. 2014 Aug 14;8(8):e3086. doi: 10.1371/journal.pntd.0003086 (PMC4133233; doi:10.1371/journal.pntd.0003086)
Supplement: Table S2 — Summary of direct PCR sequencing patient isolates for haplotyping. a Pknbpxa and b Pknbpxb gene fragments. (PDF) [file pntd.0003086.s011.pdf]

Table S2a

Summary of direct PCR sequencing patient isolates for *Pknbpxa* haplotyping.

* Sequencing results were not obtained from 7 isolates and 2 sequences were of poor quality.

Table S2b Summary of direct PCR sequencing patient isolates for *Pknbpxb*

Pknbpxa fragment amplification for direct PCR sequencing with primer *Pknbpxa* F11	Number
Primer pair *Pknbpxa*F5 and 7428R1 (Phusion®)	117
Repeat 30 isolates with *Pknbpxa* primer pair Ex1F and 7428R1(Elongase®)	21
Satisfactory sequences obtained	138
Pknbpxa haplotyping sequences not obtained	9*
Total	147

haplotyping

Pknbpxb fragment amplification for direct PCR sequencing with the primer 2318F	Number
Primer pair Xb273F and Xb3430R (Phusion®)	64
Repeat Xb273F & Xb3430R (Elongase®)	70
Satisfactory sequences obtained	134
Pknbpxa haplotyping sequences not obtained	13**
Total	147

** Sequencing results not obtained from 7 isolates, 3 sequences were of poor quality and 3 appeared to indicate mixed genotype infections.
