# Supplementary material for: Disease Progression in Plasmodium knowlesi Malaria Is Linked to Variation in Invasion Gene Family Members
Source: PLoS Negl Trop Dis. 2014 Aug 14;8(8):e3086. doi: 10.1371/journal.pntd.0003086 (PMC4133233; doi:10.1371/journal.pntd.0003086)
Supplement: Table S3 — Normality tests for analysis of genetic markers of disease progression. (PDF) [file pntd.0003086.s012.pdf]

Table S3 Normality tests for analysis of genetic markers of disease progression.

Condition	Kolmogorov-Smirnov*	Normal distribution*	N=147	Observation
Fever (days) <14	0	**Fail**	145	Also failed removing the outliers
Age (years)	0.2	OK	147	
Axillary temp (°C) <41	0	**Fail**	145	Failed after removing outliers and log transformation
Log10 Systolic BP mmHg	0.2	OK	147	
Diastolic BP mmHg	0.2	OK	147	
Mean arterial BP mmHg	0.2	OK	147	
Pulse /min	0.04	**Fail**	147	QQ-plot OK
Log10 Parasites/uL	0.2	OK	143	
PCV (%) >28, <52	0.01	OK	132	Outliers removed, QQ-plot OK
Log10 WBC's (/uL)	0.2	OK	146	
Neutrophils (%)	0.2	OK	112	
Log10 Platelets/uL	0.2	OK	147	
Sodium (mmol/L) >123, <146	0.04	OK	134	Outliers removed, QQ-plot OK
Serum creatinine umol/L**	0	**Fail**	131	Also failed the log transformation
Blood urea (mmol/L)	0	**Fail**	141	QQ-plot OK
Log2 Total bilirubin	0.16	OK	130	

(umol/L)				
Log10 Alanine aminotransferase (U/L)	0.2	OK	135	
Log10 Aspartate aminotransferase (U/L) >6, <221	0.2	OK	131	
Log10 Alkaline phosphatase UL	0.2	OK	135	
Serum Albumin (g/L), <54	0.01	OK	134	QQ-plot OK
Total protein (g/L)	0.098	OK	135	
Log10 blood glucose (g/L)	0.2	OK	111	
Log10 Plasma lactate (mmol/L), <7	0.2	OK	108	
Haemoglobin (g/dL) >8, <18	0.07	OK	143	
Red blood cells/uL blood	0.2	OK	109	
Serum globulin (g/L) <48	0.05	OK	127	QQ-plot OK
Log10 IL-10 (pg/mL)	0.2	OK	143	

Using the interquartile rule outliers

* SPSS v19.0
** Serum creatinine results are not normally distributed, non-parametric tests were used.
