# Supplementary material for: Disease Progression in Plasmodium knowlesi Malaria Is Linked to Variation in Invasion Gene Family Members
Source: PLoS Negl Trop Dis. 2014 Aug 14;8(8):e3086. doi: 10.1371/journal.pntd.0003086 (PMC4133233; doi:10.1371/journal.pntd.0003086)
Supplement: Table S4 — Pknbpxa and Pknbpxb allelic groups and significant differences between alleles in markers of disease severity. (PDF) [file pntd.0003086.s013.pdf]

Table S4 *Pknbpxa* and *Pknbpxb* allelic groups and significant differences between alleles in markers of disease severity.

***Pknbpxa* group 1 (Dimorphism)**	SNP	SNP	Dimorphism	
allele i	Xa_513,A	Xa_515,C	KH273	
allele ii	Xa_513,G	Xa_515,T	KH195	
**Condition**	**p-value***	**allele i (n)**	**allele ii (n)**	
Log10 Systolic BP mmHg	0.036	61	77	
Log10 Platelets/uL	0.037	61	77	
Log10 Plasma lactate (mmol/L), <7	0.012	42	60	
Log10 IL-10 (pg/mL) <10470	0.028	60	75	
***Pknbpxa* group 2**	SNP	SNP	SNP	Dimorphism
allele i	Xa_513,A	Xa_515,C	Xa_489,G	KH273
allele ii	Xa_513,G	Xa_515,T	Xa_489,G	KH195
allele iii	Xa_513,G	Xa_515,T	Xa_489,A	KH195
**Condition**	**p-value***	**allele i (n)**	**allele ii (n)**	
Log10 Alkaline phosphatase UL	0.037	57	54	
Log10 Plasma lactate (mmol/L), <7	0.009	42	46	
Log10 IL-10 (pg/mL) <10470	0.032	60	58	
**Condition**	**p-value***	**allele i (n)**	**allele iii (n)**	
Log10 Systolic BP mmHg	0.015	61	17	
Mean arterial BP mmHg	0.021	61	17	
Log10 Alkaline phosphatase UL	0.019	57	15	
**Condition**	**p-value***	**allele ii (n)**	**allele iii (n)**	
Red blood cells/uL blood	0.025	43	14	

***Pknbpxa* group 3**	SNP	SNP	SNP	Dimorphism
allele i	Xa_513,A	Xa_515,C	Xa_566,G	KH273
allele ii	Xa_513,A	Xa_515,C	Xa_566,T	KH273
allele iii	Xa_513,G	Xa_515,T	Xa_566,G	KH195
**Condition**	***p-value**	**allele i (n)**	**allele ii (n)**	
Log10 WBC's (/uL)	0.014	41	19	
PCV (%) >28, <52	0.037	37	16	
**Condition**	***p-value**	**allele i (n)**	**allele iii (n)**	
Log10 Plasma lactate (mmol/L), <7	0.01	30	60	
**Condition**	***p-value**	**allele ii (n)**	**allele iii (n)**	
Log10 Platelets/uL	0.023	20	77	
Log10 Alkaline phosphatase UL	0.019	18	69	
PCV (%) >28, <52	0.025	16	70	
Haemoglobin (g/dL) >8, <18	0.034	19	76	
***Pknbpxa* group 4**	SNP			
allele i	Xa_639,G			
allele ii	Xa_639,A			
**Condition**	***p-value**	**allele i (n)**	**allele ii (n)**	
Log10 Parasites/uL	0.046	65	69	
Log10 Plasma lactate (mmol/L), <7	0.049	52	50	
***Pknbpxa* group 5**	SNP	SNP	SNP	Dimorphism
allele i	Xa_513,A	Xa_515,C	Xa_769,G	KH273
allele ii	Xa_513,A	Xa_515,C	Xa_769,C	KH273
allele iii	Xa_513,G	Xa_515,T	Xa_769,G	KH195
**Condition**	***p-value**	**allele i (n)**	**allele iii (n)**	
Log10 Platelets/uL	0.023	44	77	
Log10 Alkaline phosphatase UL	0.008	41	69	
Log10 Plasma lactate (mmol/L), <7	0.016	30	60	
Haemoglobin (g/dL) >8, <18	0.034	19	76	
Log10 IL-10 (pg/mL)	0.01	44	75	

***Pknbpxa* group 6**	SNP	SNP	SNP	Dimorphism
allele i	Xa_513,A	Xa_515,C	Xa_913,T	KH273
allele ii	Xa_513,G	Xa_515,T	Xa_913,T	KH195
allele iii	Xa_513,G	Xa_515,T	Xa_913,C	KH195
**Condition**	***p-value**	**allele i (n)**	**allele ii (n)**	
Log10 Alkaline phosphatase UL	0.022	57	46	
**Condition**	***p-value**	**allele i (n)**	**allele iii (n)**	
Log10 Systolic BP mmHg	0.005	61	26	
Mean arterial BP mmHg	0.022	61	26	
Log10 Parasites/uL	0.02	60	25	
Log10 Platelets/uL	0.001	61	26	
Blood urea (mmol/L)	0.003	60	25	
Log10 Plasma lactate (mmol/L), <7	0.003	42	17	
Log10 IL-10 (pg/mL)	0.003	60	25	
Fever (days) <14	0.036	61	26	
**Condition**	***p-value**	**allele ii (n)**	**allele iii (n)**	
Log10 Parasites/uL	0.04	49	25	
Log10 WBC's (/uL)	0.02	51	26	
Log10 Platelets/uL	0.018	51	26	
Blood urea (mmol/L)	0.007	48	25	
Log10 IL-10 (pg/mL)	0.01	50	25	
***Pknbpxa* group 7**	SNP	SNP	SNP	Dimorphism
allele i	Xa_513,A	Xa_515,C	Xa_936,A	KH273
allele ii	Xa_513,G	Xa_515,T	Xa_936,A	KH195
allele iii	Xa_513,G	Xa_515,T	Xa_936,G	KH195
**Condition**	***p-value**	**allele i (n)**	**allele ii (n)**	
Log10 Alkaline phosphatase UL	0.018	57	56	
Log10 Plasma lactate (mmol/L), <7	0.026	42	46	
Log10 IL-10 (pg/mL)	0.034	60	58	

***Pknbpxa* group 8**	SNP	SNP	SNP	Dimorphism
allele i	Xa_513,A	Xa_515,C	Xa_982,T	KH273
allele ii	Xa_513,G	Xa_515,T	Xa_982,G	KH195
allele iii	Xa_513,G	Xa_515,T	Xa_982,C	KH195
**Condition**	***p-value**	**allele i (n)**	**allele ii (n)**	
Log10 Plasma lactate (mmol/L), <7	0.006	42	41	
Haemoglobin (g/dL) >8, <18	0.034	58	51	
Log10 IL-10 (pg/mL)	0.03	60	50	
Axillary temp (oC) <41	0.049	60	50	
**Condition**	***p-value**	**allele i (n)**	**allele iii (n)**	
Log10 Aspartate aminotransferase (U/L) >6, <221	0.033	56	22	

***Pknbpxa* group 9**	SNP	SNP	SNP	Dimorphism
allele i	Xa_513,A	Xa_515,C	Xa_1084,C	KH273
allele ii	Xa_513,G	Xa_515,T	Xa_1084,C	KH195
allele iii	Xa_513,G	Xa_515,T	Xa_1084,G	KH195
**Condition**	***p-value**	**allele i (n)**	**allele ii (n)**	
Log10 Systolic BP mmHg	0.045	61	38	
Log10 Alkaline phosphatase UL	0.037	57	34	
Log10 Plasma lactate (mmol/L), <7	0.026	42	30	
Log10 IL-10 (pg/mL)	0.009	60	38	
**Condition**	***p-value**	**allele i (n)**	**allele iii (n)**	
Log10 Plasma lactate (mmol/L), <7	0.036	42	30	

***Pknbpxa* group 10**	SNP	SNP	SNP	Dimorphism
allele i	Xa_513,A	Xa_515,C	Xa_1102,C	KH273
allele ii	Xa_513,G	Xa_515,T	Xa_1102,C	KH195
allele iii	Xa_513,G	Xa_515,T	Xa_1102,G	KH195
**Condition**	***p-value**	**allele i (n)**	**allele ii (n)**	
Log10 Plasma lactate (mmol/L), <7	0.043	42	27	
**Condition**	***p-value**	**allele i (n)**	**allele iii (n)**	
Log10 Systolic BP mmHg	0.009	61	42	
Diastoloc BP mmHg	0.09	61	42	
Mean arterial BP mmHg	0.021	61	42	
Log10 Platelets/uL	0.024	61	42	
Log10 Alkaline phosphatase UL	0.017	57	37	
Blood urea (mmol/L)	0.037	60	40	
Log10 Plasma lactate (mmol/L), <7	0.022	42	33	
Haemoglobin (g/dL) >8, <18	0.029	58	41	
***Pknbpxa* group 11**	SNP	SNP	SNP	Dimorphism
allele i	Xa_513,A	Xa_515,C	Xa_1171,A	KH273
allele ii	Xa_513,G	Xa_515,T	Xa_1171,A	KH195
allele iii	Xa_513,G	Xa_515,T	Xa_1171,G	KH195
**Condition**	***p-value**	**allele i (n)**	**allele ii (n)**	
Log10 Platelets/uL	0.035	61	50	
Log10 Alkaline phosphatase UL	0.01	57	44	
Log10 Plasma lactate (mmol/L), <7	0.006	42	36	
Log10 IL-10 (pg/mL)	0.015	60	49	
**Condition**	***p-value**	**allele i (n)**	**allele iii (n)**	
Log10 Systolic BP mmHg	0.034	61	27	
Diastoloc BP mmHg	0.016	61	27	
Mean arterial BP mmHg	0.018	61	27	
Fever (days) <14	0.021	61	27	
**Condition**	***p-value**	**allele ii (n)**	**allele iii (n)**	
Sodium (mmol/L) >123, <146	0.038	45	24	
Fever (days) <14	0.028	48	27	

***Pknbpxa* group 12**	SNP	SNP	SNP	Dimorphism
allele i	Xa_513,A	Xa_515,C	Xa_1281,A	KH273
allele ii	Xa_513,G	Xa_515,T	Xa_1281,A	KH195
allele iii	Xa_513,G	Xa_515,T	Xa_1281,G	KH195
**Condition**	***p-value**	**allele i (n)**	**allele ii (n)**	
Log10 Alkaline phosphatase UL	0.03	57	52	
Log10 Plasma lactate (mmol/L), <7	0.012	42	47	
**Condition**	***p-value**	**allele i (n)**	**allele iii (n)**	
Diastoloc BP mmHg	0.023	61	20	
Mean arterial BP mmHg	0.028	61	20	
Log10 Platelets/uL	0.015	57	20	
Log10 Alkaline phosphatase UL	0.033	57	17	
Log10 IL-10 (pg/mL)	0.038	60	20	
Fever (days) <14	0.014	61	19	
**Condition**	***p-value**	**allele i (n)**	**allele iii (n)**	
Fever (days) <14	0.018	56	19	

***Pknbpxb* group 1**	SNP	SNP				
allele i	Xb_2637,A	Xb_2638,C				
allele ii	Xb_2637,C	Xb_2638,A				
**Condition**	***p-value**	**allele i (n)**	**allele ii (n)**			
Haemoglobin (g/dL) >8, <18	0.002	111	20			
Axillary temp (oC) <41	0.002	112	20			
***Pknbpxb* group 2**	SNP	SNP	SNP	SNP	SNP	SNP
allele i	Xb_2711,C	Xb_2740,A	Xb_2757,A	Xb_2802,G	Xb_2834,C	Xb_3115,G
allele ii	Xb_2711,T	Xb_2740,G	Xb_2757,G	Xb_2802,A	Xb_2834,A	Xb_3115,A
allele iii	Xb_2711,T	Xb_2740,G	Xb_2757,G	Xb_2802,A	Xb_2834,A	Xb_3115,G
**Condition**	***p-value**	**allele i (n)**	**allele ii (n)**			
Haemoglobin (g/dL) >8, <18	0.033	50	47			
Log10 WBC's (/uL)	0.001	50	48			
PCV (%) >28, <52	0.013	47	44			
**Condition**	***p-value**	**allele i (n)**	**allele iii (n)**			
Log10 Aspartate aminotransferase (U/L) >6, <221	0.002	47	24			

Log2 Total Bilirubin umol/L	0.043	47	23

**Condition**	***p-value**	**allele ii (n)**	**allele iii (n)**
Log10 Parasites/uL	0.022	47	30
Log10 Plasma lactate (mmol/L), <7	0.005	39	23
