# Supplementary material for: Disease Progression in Plasmodium knowlesi Malaria Is Linked to Variation in Invasion Gene Family Members
Source: PLoS Negl Trop Dis. 2014 Aug 14;8(8):e3086. doi: 10.1371/journal.pntd.0003086 (PMC4133233; doi:10.1371/journal.pntd.0003086)
Supplement: Table S5 — Reference isolates used for generating full-length (8501 bp) Pknbpxa and (3506 bp) Pknbpxb gene sequences. (PDF) [file pntd.0003086.s014.pdf]

Table S5. Reference isolates used for generating full-length (8501bp) *Pknbpxa* and (3506bp) *Pknbpxb* gene sequences

Isolate	Collection site	Collection Date	Parasites/uL	*Pknbpxa* Accession Numbers	*Pknbpxb* Accession Numbers
1) KH195	Kapit	20/03/02	2240	KF186569	KF186574
2) KH229	Kapit	29/06/03	4800	KF186568	KF186573
3) KH273	Kapit	25/07/03	3000	KF186570	KF186575
4) SKS047	Sarikei	09/02/08	10000	KF186571	KF186576
5) SKS371	Sarikei	05/10/10	6640	KF186572	KF186577

P. knowlesi reference isolates from patients recruited at different periods and geographically distinct locations in the Kapit and Sarikei districts, Sarawak. 'KH' samples were taken from a previous study with consent to use in future studies [1] and 'SKS' from the present study.

1. Lee KS, Divis PC, Zakaria SK, Matusop A, Julin RA, et al. Plasmodium knowlesi: Reservoir Hosts and Tracking the Emergence in Humans and Macaques. PLoS Pathog 7: e1002015.
