# Supplementary material for: Disease Progression in Plasmodium knowlesi Malaria Is Linked to Variation in Invasion Gene Family Members
Source: PLoS Negl Trop Dis. 2014 Aug 14;8(8):e3086. doi: 10.1371/journal.pntd.0003086 (PMC4133233; doi:10.1371/journal.pntd.0003086)
Supplement: Table S6 — P. knowlesi polymorphisms in the haplotyping fragment (885 bp) making up the Pknbpxa dimorphism. (PDF) [file pntd.0003086.s015.pdf]

Table S6. *P. knowlesi* polymorphisms in the haplotyping fragment (885bp) making up the *Pknbpxa* dimorphism.

***Pknbpxa* dimorphism**	**(S) 513**	**(NS) 515**	**(NS) 678**	**(NS) 683**	**(NS) 689**	**(NS) 718**	**(NS) 750**	**(NS) 751**	**(NS)757**	**(NS)758**
**KH273**	**A**	**C**	**T**	**G**	**C**	**C**	**T**	**A**	**G**	**A**
**KH195**	**G**	**T**	**G**	**T**	**G**	**G**	**G**	**G**	**C**	**C**

**Pknbpxa dimorphism (cont'd)**	**(NS)763**	**(NS)764**	**(NS) 766**	**(NS) 772**	**(NS) 773**	**(NS) 958**	**(NS) 971**	**(NS) 1027**	**(NS) 1057**	**(NS) 1094**
**KH273**	**A**	**C**	**A**	**G**	**G**	**C**	**A**	**T**	**G**	**G**
**KH195**	**G**	**T**	**T**	**A**	**A**	**T**	**C**	**C**	**A**	**A**

**Pknbpxa dimorphism (cont'd)**	**(NS) 1120**	**(S) 1203**	**(NS) 1268**	**(S) 1284**	**(NS) 1297**	**(NS) 1310**	**(NS) 1318**	**(S) 1323**	**(NS) 1328**
**KH273**	**G**	**G**	**A**	**T**	**C**	**A**	**T**	**T**	**T**
**KH195**	**A**	**C**	**C**	**C**	**A**	**G**	**A**	**C**	**G**

The number is the nucleotide position relative to EU867791. Non-synonymous (NS) and synonymous (S) substitutions.
