# Supplementary material for: Disease Progression in Plasmodium knowlesi Malaria Is Linked to Variation in Invasion Gene Family Members
Source: PLoS Negl Trop Dis. 2014 Aug 14;8(8):e3086. doi: 10.1371/journal.pntd.0003086 (PMC4133233; doi:10.1371/journal.pntd.0003086)
Supplement: Table S7 — Non-synonymous sites and amino acid changes in P. knowlesi Pknbpxa and Pknbpxb haplotyping fragments. a Pknbpxa 138 patient isolates, b Pknbpxb 134 patient isolates. (PDF) [file pntd.0003086.s016.pdf]

Table S7a Non-synonymous sites and amino acid changes in the *P. knowlesi Pknbpxa* haplotyping fragment from 138 patient isolates.

										1	1	1	1	1	1	1	1	1	1	1	1	1	1	1	1	1	1	2	2	2	2	2	2	2	2	2	2	2	2	2	2	2	2	2	3	3	3	3	3	3	3	3	3
5	5	6	6	6	6	7	8	8	9	0	0	2	2	2	3	3	3	4	4	4	5	5	5	5	9	9	9	0	0	1	1	1	2	2	3	3	4	5	6	6	6	6	8	9	0	1	1	2	3	3	3	3	4
4	8	0	1	5	7	8	0	4	7	5	7	1	3	5	1	5	7	5	6	8	0	1	2	3	0	8	9	0	2	0	5	9	3	8	7	8	8	7	0	3	4	9	6	3	2	3	8	8	1	2	5	8	4
P	R	N	L	S	F	T	S	G	H	R	P	L	I	G	A	V	G	K	V	P	V	L	D	N	R	D	N	P	D	D	Y	A	A	L	M	P	R	Q	D	E	V	N	D	M	I	K	A	I	V	S	T	W	S
.	.	.	.	.	.	.	.	.	.	.	.	.	.	.	.	.	.	.	.	.	.	.	.	.	.	.	Y	S	N	.	.	.	.	.	.	.	.	.	.	.	.	.	.	.	.	.	.	.	.	.	.	.	.
.	.	.	.	.	.	.	.	.	.	.	.	.	.	.	.	.	.	.	.	.	.	.	.	.	.	.	.	.	.	.	.	.	.	.	.	.	.	.	.	.	.	.	N	.	.	.	.	.	.	.	.	.	.
.	.	.	.	.	.	A	.	.	.	.	.	.	.	.	.	.	.	.	.	.	.	.	.	.	.	.	.	.	.	.	.	.	.	.	.	.	.	E	.	.	.	.	N	.	.	.	.	.	.	.	.	.	.
.	.	.	.	.	.	.	.	.	.	.	.	.	.	.	.	.	.	.	.	.	.	.	.	.	.	.	.	S	.	.	.	.	.	.	.	.	.	.	.	.	I	.	N	K	V	.	.	.	.	.	.	.	.
.	.	.	.	.	.	.	.	.	.	.	.	.	.	.	.	.	.	.	.	.	.	.	.	.	.	.	.	S	.	.	.	.	.	.	.	.	.	.	.	.	.	.	N	.	.	.	.	.	.	.	.	.	.
.	.	.	.	.	.	.	.	.	.	.	.	.	.	.	.	.	.	.	.	.	.	.	.	.	.	.	.	S	E	.	.	.	P	.	.	.	.	.	.	.	.	.	.	.	.	.	.	.	.	.	.	.	.
.	.	.	.	.	.	.	.	.	.	.	.	.	.	.	.	.	.	.	.	.	.	.	.	.	.	.	.	S	G	.	.	.	.	.	.	.	.	E	.	Q	.	.	N	.	.	.	.	.	.	.	.	.	.
.	.	.	.	.	.	.	.	.	.	.	.	.	.	.	.	.	.	.	.	.	.	.	.	.	.	.	.	.	.	.	.	.	P	M	.	.	.	E	.	Q	.	.	N	.	.	.	.	.	.	.	.	.	.
.	.	.	.	.	.	.	.	.	.	.	.	.	.	.	.	.	.	.	.	.	.	.	.	.	S	.	.	S	N	N	.	.	.	.	.	.	.	E	.	Q	.	.	.	.	.	.	.	.	.	.	.	.	.
.	.	.	.	.	.	.	.	.	.	.	.	.	.	.	.	.	V	.	.	.	.	.	.	.	.	.	.	.	.	.	.	.	.	.	.	.	.	E	.	Q	.	.	N	.	.	E	.	.	.	.	.	.	.
.	.	.	.	.	.	.	.	.	.	.	.	.	.	.	T	.	.	.	.	.	.	.	.	.	.	.	.	S	.	.	.	.	.	.	.	.	.	E	.	Q	.	.	N	.	.	.	.	.	.	.	.	.	.
.	.	.	.	.	.	.	R	.	.	.	.	.	.	.	.	.	.	.	.	.	.	.	.	.	.	.	.	.	.	.	.	.	.	.	.	.	.	.	.	.	.	.	N	.	.	.	.	.	.	.	.	.	.
.	.	.	.	.	.	.	.	.	Y	.	.	.	.	.	.	.	.	.	.	.	.	.	.	.	.	.	.	S	.	.	.	.	P	.	.	.	.	E	.	Q	.	.	N	.	.	.	.	.	.	.	.	.	.
.	.	.	.	I	.	.	.	.	.	.	.	.	.	.	.	.	.	.	.	.	.	.	.	.	.	.	.	S	.	.	.	.	.	.	.	.	.	E	.	Q	.	.	N	.	.	.	.	.	.	.	.	.	.
.	K	.	.	.	.	.	.	.	.	.	.	.	.	.	.	.	.	.	.	.	.	.	.	.	.	.	.	S	.	.	.	.	.	.	.	.	.	E	.	Q	.	.	N	.	.	.	.	.	.	.	.	.	.
.	.	.	.	.	.	.	.	.	.	.	.	.	.	.	.	.	.	.	.	.	.	.	.	.	.	.	.	S	N	.	.	.	.	.	.	.	.	.	.	.	.	.	N	.	.	E	.	.	.	.	.	.	.
.	.	.	.	.	.	.	.	.	.	.	.	.	.	.	.	.	.	.	.	.	.	.	.	.	.	.	.	.	.	.	.	.	.	.	.	.	.	.	.	.	I	.	N	.	.	.	.	.	.	.	.	.	.
.	.	.	.	.	.	.	.	.	.	.	.	.	.	.	.	.	.	.	.	.	.	.	.	.	.	.	.	.	.	.	.	.	P	.	.	.	.	E	.	Q	.	.	.	.	.	.	.	.	.	.	.	.	.
.	.	.	.	.	.	.	.	.	.	.	.	.	.	.	.	.	.	.	.	.	.	.	.	.	.	.	.	.	.	.	.	.	.	.	.	.	.	.	.	.	.	.	.	.	.	.	.	.	.	.	.	.	.
R	.	.	.	.	.	.	.	.	.	.	.	.	.	.	.	.	.	.	.	.	.	.	.	.	.	.	.	S	.	.	.	.	.	.	.	.	.	E	.	Q	.	.	N	.	.	E	.	.	.	.	.	.	.
.	.	.	.	.	.	.	.	.	.	.	.	.	.	.	.	.	.	.	.	.	.	.	.	.	.	.	.	.	.	.	.	.	.	.	.	.	.	E	.	Q	.	.	N	K	.	.	.	.	.	.	.	.	.
.	.	.	.	.	.	.	.	.	.	.	.	.	.	.	.	.	.	.	.	.	.	.	.	.	.	.	.	.	.	.	.	.	P	.	.	.	.	.	.	.	.	.	.	.	.	.	.	.	.	.	.	.	.
.	.	.	.	.	.	.	.	.	.	.	.	.	.	.	.	.	.	.	.	.	.	.	.	.	.	.	.	S	.	.	.	.	.	.	.	.	.	.	.	Q	.	.	.	.	.	.	.	.	.	.	.	.	T
.	.	.	F	.	.	.	.	.	.	.	.	.	.	.	.	.	.	.	.	.	.	.	.	.	.	.	.	S	.	.	.	.	P	.	.	.	.	E	.	Q	.	.	.	.	.	.	.	.	.	.	.	.	.
.	.	.	.	.	S	.	.	V	.	.	.	F	R	A	.	L	.	N	M	D	T	I	.	G	.	.	.	S	.	.	H	D	S	.	.	S	G	.	G	Q	.	D	N	.	.	.	D	L	.	N	S	L	.
.	.	.	.	.	S	.	.	V	.	.	.	F	R	A	.	L	.	N	M	D	T	I	.	G	.	.	.	S	.	.	H	D	S	.	.	S	G	.	G	Q	.	D	N	.	.	.	D	L	.	N	S	L	.
.	.	.	.	.	S	.	.	.	.	.	.	F	R	A	.	L	.	N	M	D	T	I	.	G	.	.	.	S	.	.	H	D	S	.	.	S	G	.	G	Q	.	D	N	.	.	.	D	L	.	N	S	L	.
.	.	.	.	.	S	.	.	V	.	.	.	F	R	A	.	L	.	N	M	D	T	I	.	G	.	.	.	S	.	.	H	D	S	.	I	S	G	.	G	Q	.	D	N	.	.	.	D	L	.	N	S	L	.
.	.	.	.	.	S	.	.	V	.	.	.	F	R	A	.	L	.	N	M	D	T	I	.	G	.	.	.	S	.	.	H	D	S	.	.	S	G	.	G	Q	.	D	N	.	.	.	D	L	.	N	S	L	.
.	.	.	.	.	S	.	.	V	.	.	.	F	R	A	.	L	.	N	M	D	T	I	.	G	.	.	.	S	.	.	H	D	S	.	.	S	G	.	G	Q	.	D	N	.	.	.	D	L	.	N	S	L	.
.	.	I	.	.	S	.	.	.	.	.	.	F	R	A	.	L	.	N	M	D	T	I	H	G	.	.	.	S	.	.	H	D	S	.	.	S	G	.	G	Q	.	D	N	.	.	.	D	L	.	N	S	L	.
.	.	.	.	.	S	.	.	.	.	.	.	F	R	A	.	L	.	N	M	D	T	I	.	G	.	N	.	S	.	.	H	D	S	.	.	S	G	.	G	Q	.	D	N	.	.	.	D	L	L	N	S	L	.

Dots indicate identical amino acids. Codon positions are represented vertically and codon position numbering based on Pknbpxa (ACJ54535) sequence.

Table S7b Non-synonymous sites and amino acid changes in the *P. knowlesi Pknbpxb* haplotyping fragment from 134 patient isolates.

685	693	697	704	713	717	728	730	740	747	780	783	807	817	818	826	847	848	859	921	928	937	940	942	947	953
E	S	L	I	I	N	K	N	A	Q	H	Q	T	I	F	K	I	T	T	L	T	E	N	E	H	Q
D	.	.	.	.	.	.	.	.	.	N	.	.	.	.	.	.	.	.	.	.	.	.	K	.	.
D	.	.	.	.	.	.	.	.	.	N	.	.	.	.	.	M	.	.	.	.	.	.	K	.	.
D	.	.	.	F	.	.	.	.	.	N	.	.	.	.	.	.	.	.	.	.	.	.	K	.	H
D	.	.	.	.	.	.	.	.	.	N	.	.	.	.	.	M	.	.	.	.	.	.	K	.	.
D	.	.	.	.	.	.	.	.	.	.	.	.	.	.	.	.	.	.	.	.	.	.	K	.	.
.	.	.	.	.	.	.	.	.	.	.	.	.	.	.	.	.	.	.	.	.	K	.	.	L	.
D	.	.	.	.	.	.	D	.	.	.	K	M	V	.	.	.	N	.	.	.	.	.	K	.	.
D	.	.	M	.	.	.	.	.	.	.	.	M	V	.	.	.	N	.	.	.	.	.	.	.	.
D	.	.	.	.	.	.	.	.	.	N	.	M	V	.	.	.	N	.	.	.	.	.	.	.	.
D	.	.	.	.	.	.	.	.	.	.	.	M	V	.	.	.	N	.	.	.	.	.	.	.	.
D	N	.	.	.	.	.	.	.	.	.	K	M	V	.	.	.	N	.	.	.	.	.	K	.	.
D	.	S	.	.	.	E	.	.	.	N	.	M	V	.	.	.	N	.	.	.	.	S	K	.	.
D	.	.	.	.	.	.	.	.	.	.	.	M	V	.	.	.	N	.	.	.	.	.	K	.	.
D	.	.	M	.	.	.	.	S	.	D	.	M	V	.	.	.	N	.	.	.	.	.	K	.	.
D	.	.	.	.	.	.	.	.	L	.	K	M	V	.	.	.	N	.	.	.	.	.	K	.	.
D	.	.	.	.	.	.	.	.	.	N	.	M	V	.	E	.	N	.	.	.	.	.	.	.	.
D	.	.	.	.	.	.	.	.	.	.	.	M	V	.	.	.	N	.	.	.	.	.	K	.	.
D	.	.	.	.	.	.	.	.	.	.	.	M	V	.	.	.	N	.	.	.	.	.	.	.	.
D	.	.	M	.	.	.	.	.	.	.	.	M	V	.	.	.	N	.	.	.	.	.	K	.	.
D	.	.	.	.	.	.	.	.	.	.	.	M	V	.	.	.	N	.	.	.	.	S	K	.	.
D	.	.	.	.	.	.	D	.	.	.	K	M	V	.	.	.	N	.	.	I	.	.	.	.	.
D	.	.	.	.	.	.	.	.	.	.	.	.	.	.	.	.	N	.	.	.	.	.	.	.	.
D	.	.	.	.	.	.	D	.	.	.	K	M	V	.	.	.	.	.	I	.	.	S	K	.	.
D	.	.	.	.	K	.	.	.	.	.	.	M	V	.	.	.	N	S	.	.	.	.	K	.	.
D	.	.	.	.	.	.	D	.	.	.	.	.	.	L	.	.	N	.	.	.	.	.	K	.	.

Dots indicate identical amino acids. Codon positions are represented vertically and codon position numbering based on Pknbpxb (ACJ54536) amino acid sequence.
