# Supplementary material for: Disease Progression in Plasmodium knowlesi Malaria Is Linked to Variation in Invasion Gene Family Members
Source: PLoS Negl Trop Dis. 2014 Aug 14;8(8):e3086. doi: 10.1371/journal.pntd.0003086 (PMC4133233; doi:10.1371/journal.pntd.0003086)
Supplement: Text S1 — Additional information on patient recruitment, blood sample processing and the study cohort. (DOCX) [file pntd.0003086.s017.docx]

**Supplementary Text**

Text **S1** Additional information on patient recruitment, blood sample processing and the study cohort.

**Patient recruitment**

Study recruitment packs were provided at each site. Each pack contained patient information sheets in two languages (English and Bahasa Malaysia), consent forms in two languages, a short patient history form, blood collection material required and a study flow chart. The study inclusion criteria was microscopy positive malaria diagnosis, >15 years, not pregnant, no other comorbidity and not receiving antimalarial treatment. In addition PCR-confirmation of single Plasmodium species infection was required. PCR assays were performed retrospectively therefore all patients microscopically positive for malaria parasites fulfilling all but the PCR criterion were recruited. The study team at each site consisted of the laboratory staff responsible for routine diagnostic malaria microscopy, nursing and clinical staff on the male and female adult medical wards and high dependency and intensive care wards. The members of the team were trained in the recruitment procedure with regular updates and feedback sessions. Briefly, the malaria microscopist was responsible for informing the attending clinical staff when a patient was malaria positive by routine microscopy. Informed consent was obtained from the patient or responsible relative before collecting 10mL of pre-treatment blood. The blood sample was divided between the blood collection tubes provided. The patient was then treated and cared for following normal hospital procedure. The study protocol was designed to facilitate patient recruitment before treatment with minimum delay in patient care and undue burden on health care professionals.

**Blood sample processing**

The pre-treatment blood sample was divided between tubes as follows: EDTA for parasite DNA analyses (3mL), fluoride oxalate for plasma for glucose and lactate levels (2mL) and a clotted sample (5mL) for assays requiring serum. A small quantity of the EDTA sample (approx. 25uL) was spotted onto filter paper and dried for PCR confirmation of infecting Plasmodium species. Serum was used for liver and renal function tests on site and the balance labelled and stored frozen in Cryovials (Nunc®) along with the EDTA whole blood and plasma samples. The samples were stored frozen on site and transported at sub-zero temperatures to the Malaria Research Centre, University Malaysia Sarawak, (UNIMAS) Kuching at regular intervals during the study. DNA amplification, cloning and sequencing were conducted in UNIMAS and serum and plasma samples were shipped on dry ice to St George’s University of London for glucose, lactate and IL-10 assays.

**Supplementary results**

*Patient cohort*

In total 389 patients were admitted with microscopy confirmed malaria at Sibu and Sarikei hospitals between January 2008 and February 2011. Of these,175 patients (45%) were recruited at Sibu Hospital and 214 (55%) at Sarikei Hospital. Forty three patients were excluded following PCR; 29 were PCR negative, two had missing samples and 12 patients had mixed species infections. Of the remaining 346 patients, 304 had single species infections (figures S1a and S1b).
